# Optimising environmental factors for maximal lactate productivity in *Synechocystis* sp. PCC 6803 through a design of experiments approach

**DOI:** 10.1186/s13068-025-02718-2

**Published:** 2025-11-25

**Authors:** Matthew Faulkner, Fraser Andrews, Nigel S. Scrutton

**Affiliations:** Manchester Institute of Biotechnology and Department of Chemistry, The University of Manchester, 131 Princess Street, Manchester, M1 7DN UK

**Keywords:** Synechocystis sp. PCC 6803, Lactate, Photosynthesis, Bioproduction, Design of experiments, Optimisation, Environmental factors

## Abstract

Genetic and metabolic engineering of *Synechocystis* sp. PCC 6803 has given rise to strains that produce a variety of bio-chemicals. Despite these successes, improvements in productivity metrics are required to achieve economically viable bio-production of carbon dioxide-derived compounds. Previously, environmental factors have been leveraged to increase product yields. Here, we optimise multiple environmental factors simultaneously using Design of Experiments (DOE) principles and find conditions that maximise L-lactate production. Light intensity, glycerol concentration, and light–dark cycle were found to be significant factors. Optimising these conditions resulted in a 6.3- and 7.4-fold increase in titre and yield. The results detailed here could have implications for metabolic engineering of, and bioprocesses using, *Synechocystis* sp. PCC 6803. In the future, DOE-mediated optimisation of environmental conditions could effectively maximise product titres from different production strains, or the enhanced conditions described here could be directly implemented in other metabolic engineering projects.

**Supplementary Information:**

The online version contains supplementary material available at 10.1186/s13068-025-02718-2.

## Introduction

For cyanobacterial biotechnology to become commercially viable, bio-productivities and yields need to be improved [1]. Metabolic engineering has been widely implemented to streamline metabolism and increase productivity metrics. Despite its successful track record, the process of genetic engineering is slow, fickle, and limited to a small number of modifications, usually deployed one at a time [2]. Metabolic engineering often does not exploit wholesale changes in cyanobacterial physiology, unless regulatory genes, such as sigma factors are targeted [3]. Speaking to this idea, a recent review discussed the merit of expanding the remit of metabolic engineering to encompass environmental stress engineering to harness untapped gains in productivity [4].

To leverage wholesale physiological changes for bio-product formation, environmental factors, such as nitrogen and phosphorus depletion [5, 6], excess light and carbon [4, 7, 8], and light–dark cycles [9] have been employed to increase bio-product titres [10]. Often one stress is applied at a time [4], though combining nitrogen depletion and high light induces a metabolic overflow response in ∆*glgC Synechocystis* sp. PCC 6803, demonstrating the efficacy of combining environmental factors [11]. Currently, there is an absence of knowledge surrounding the optimisation of multiple environmental stresses to increase titres.

Many analytical methodologies could study the effect of environmental factors on pyruvate-derived product synthesis in *Synechocystis* sp. PCC 6803 including, one factor at a time (OFAAT) optimisation, Bayesian optimisation, and design of experiments approaches (DOE). Each of these has its advantages and disadvantages. For example, OFAAT is slow and ignores relationships between factors. Bayesian optimisation finds interactions between factors but requires sequential rounds of optimisation each informing a model [12, 13]. To maintain the model accuracy over multiple rounds, it would be paramount to eliminate experimental noise, biological variability, and implement absolute experimental repeatability, all tall tasks when working with *Synechocystis* sp. PCC 6803. In this study, DOE principles were chosen to fill this knowledge gap as they offer experimental minimisation and can fully describe interactions between factors in a manner compatible with physiological interpretation [14].

Various DOE methodologies allow the screening and optimisation of multiple factors simultaneously to maximise or minimise output variables and determine complex interactions between factors [14]. Mathematical models are created and fitted to the data, facilitating the description of output variables by input factors and allowing subsequent optimisation [14]. The power of this approach is well documented with increased biomass and bio-product yields in a range of microorganisms [15]. In the context of cyanobacterial biotechnology, this technique has been limited to optimising the environmental parameters: temperature, light, bicarbonate, and exogenous alendronate addition for isoprene production in *Synechococcus* UTEX 2973 [16] as well as phosphate, nitrate, carbon and light for the production of citramalate in *Synechocystis* sp. PCC 6803 [7].

**Fig. 1:**
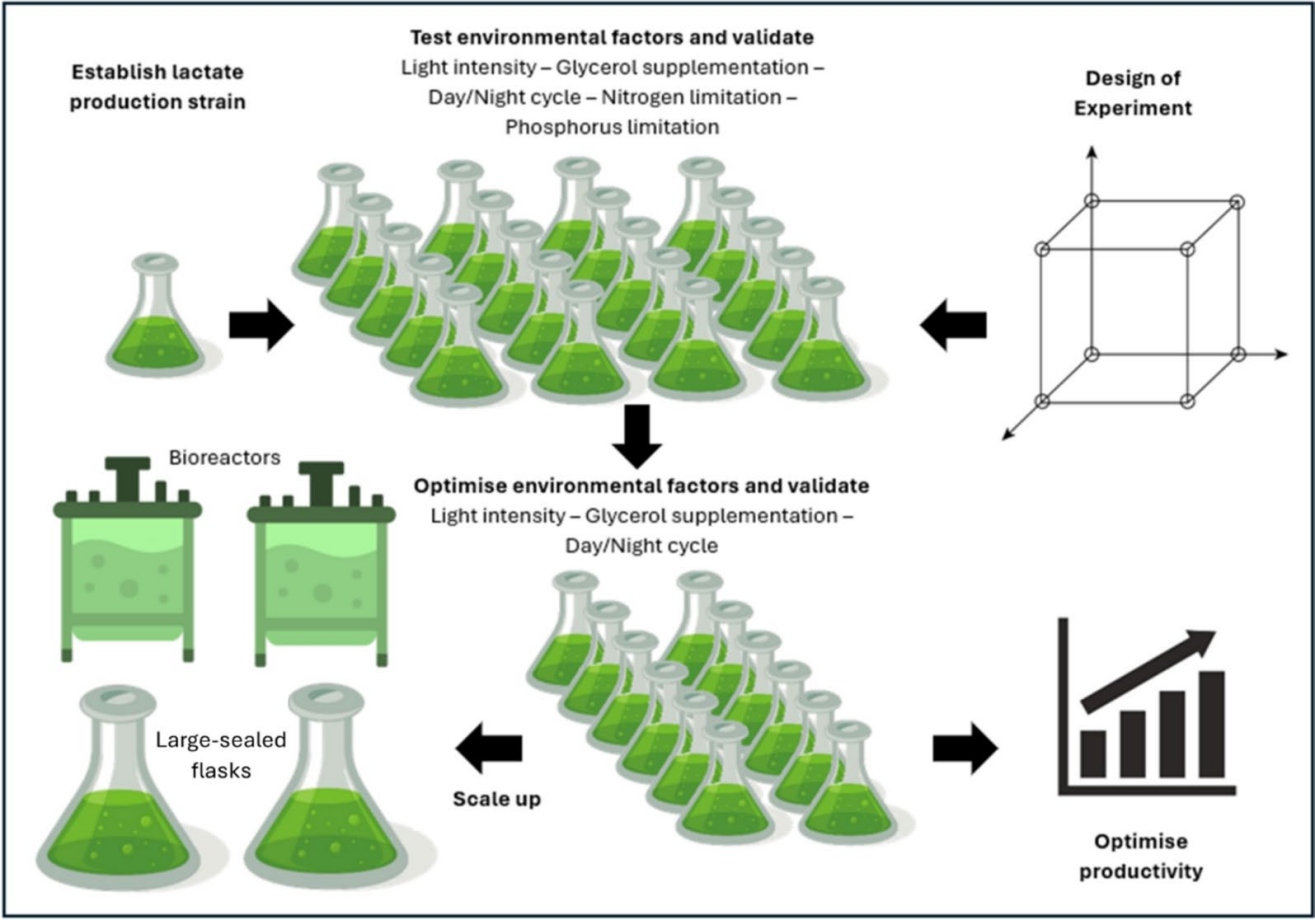
Overview of the experimental workflow in this study. Step 1, top left, create the lactate production strain. Step 2, top centre, use DOE to validate the strain’s production capacity and determine environmental factors to which the productivity of lactate is sensitive. Step 3, bottom centre, repeat the DOE investigating only those parameters found to have a significant effect on productivity. Step 4, bottom left, scale up and compare lactate productivity in 2-L bioreactors and flasks

This study uses DOE principles to optimise multiple environmental factors, and the interaction between factors, for maximal L-lactate production in *Synechocystis* sp. PCC 6803 (Fig. 1). L-Lactate synthesis was chosen for two reasons. Firstly, lactic acid can be polymerised to form poly(lactic acid), a strong, biocompatible and biodegradable bioplastic [17]. Secondly, it is a derivative of pyruvate, a core metabolite used for the synthesis of many bio-products. DOE was performed to increase L-lactate titres to uncover synergistic combinations of environmental factors that may be applied to similar pyruvate-derived products. This approach could help improve yields in other cyanobacterial metabolic engineering projects by creating new knowledge on how the optimisation of stresses impacts productivity. This may be an important step that may help raise Titre, Rate, and Yield (TRY) metrics and realise a cyanobacterial biotechnology industry [18].

## Results

### Testing the reproducibility of DOE methodology

An engineered strain of *Synechocystis* sp. PCC 6803 was constructed by genome integration of a L-lactate dehydrogenase gene (Material and methods) and used to produce L-lactate photosynthetically from CO_2._ Subsequently, L-lactate yield was optimised through a series of iterative design of experiments and validated by repeating the apparent optimum in 2-L photobioreactors (Fig. 1).

Experiments suggested that the loss of HCO_3_^− ^via evolution to CO_2_, limited growth in open flasks, thus a sealed flask system was adopted which improved growth (Supplementary Fig. 1). A buffer to HCO_3_^−^ ratio that controlled pH and yielded a high density of cells was identified (Supplementary Fig. 2). After 4 days, growth in this closed system was limited by HCO_3_^−^ availability (Supplementary Fig. 3 and Supplementary Fig. 4). To investigate light intensity and light–dark cycle changes, a bespoke light control platform was constructed (Supplementary Fig. 5). It was observed that the final biomass measurements were unaffected by light intensity, as different light intensities did not have a statistically significant impact on the final biomass yields of closed flasks with 80 mM HCO_3_^− ^and 200 mM HEPES (Supplementary Fig. 6)**.** The resulting system facilitated pH control, fast growth, and high biomass accumulation in a small flask. It was postulated that these metrics would translate into higher carbon fixation rates and greater L-lactate productivity. This could aid statistical resolution as low L-lactate production rates, limited by carbon fixation, could obfuscate the effects of changing input factor levels (e.g. amount of nitrogen) and curtail the accuracy of data analysis.

This study elected to use DOE principles due to its experimental efficiency and analytical power. To verify that experimental data points collected using the DOE methodology would be reproducible, a small-scale factor screening experiment was performed (Supplementary Fig. 7). At each data point, select environmental factors were changed and the experiment was performed in duplicate to assess the repeatability of the scientific method. Following an initial growth phase of 4 days, cultures were diluted to an OD _720 nm_ of 1.0 in a new medium where 0.88 mM (5%) nitrogen, no phosphorus, and high light conditions were differentially applied (Supplementary Fig. 7.A). Following 7 days of incubation, OD _720 nm_, L-lactate titre, and L-lactate yield (final titre normalised by OD _720 nm_) were measured (Supplementary Fig. 7). In this study, we define titre as the total accumulation of L-lactate in g L^−1^, yield as the titre per biomass (g_(L-lactate)_ L^−1^ g_(biomass)_ L^−1^), and productivity as the yield per unit time (g_(L-lactate)_ L^−1^ g_(biomass)_ L^−1^ h^−1^).

Two-way analysis of variance (ANOVA) suggested that culture conditions accounted for 95.72, 98.08, and 99.16% of variance, and were the significant factor (*p* = 0.0009, 0.0002, 0.0001) for final OD _720 nm_, L-lactate titre and L-lactate yield, respectively (Supplementary Fig. 7). Conversely, variance within repeats was not deemed a significant factor impacting output variables (*p* = 0.2147, 0.6912, 0.2481, respectively). This validates that changing culture conditions provides sufficient variance in all measured outputs for input factors to overcome experimental noise. This supported the experimental and statistical methodology and encouraged the performance of a large-scale DOE. All conditions with no phosphorus performed poorly in all metrics; accordingly, the factor level was increased to 1.15 µM phosphorus (5% of standard BG11) for future experiments.

### Identification of factors affecting L-lactate productivity

**Fig. 2:**
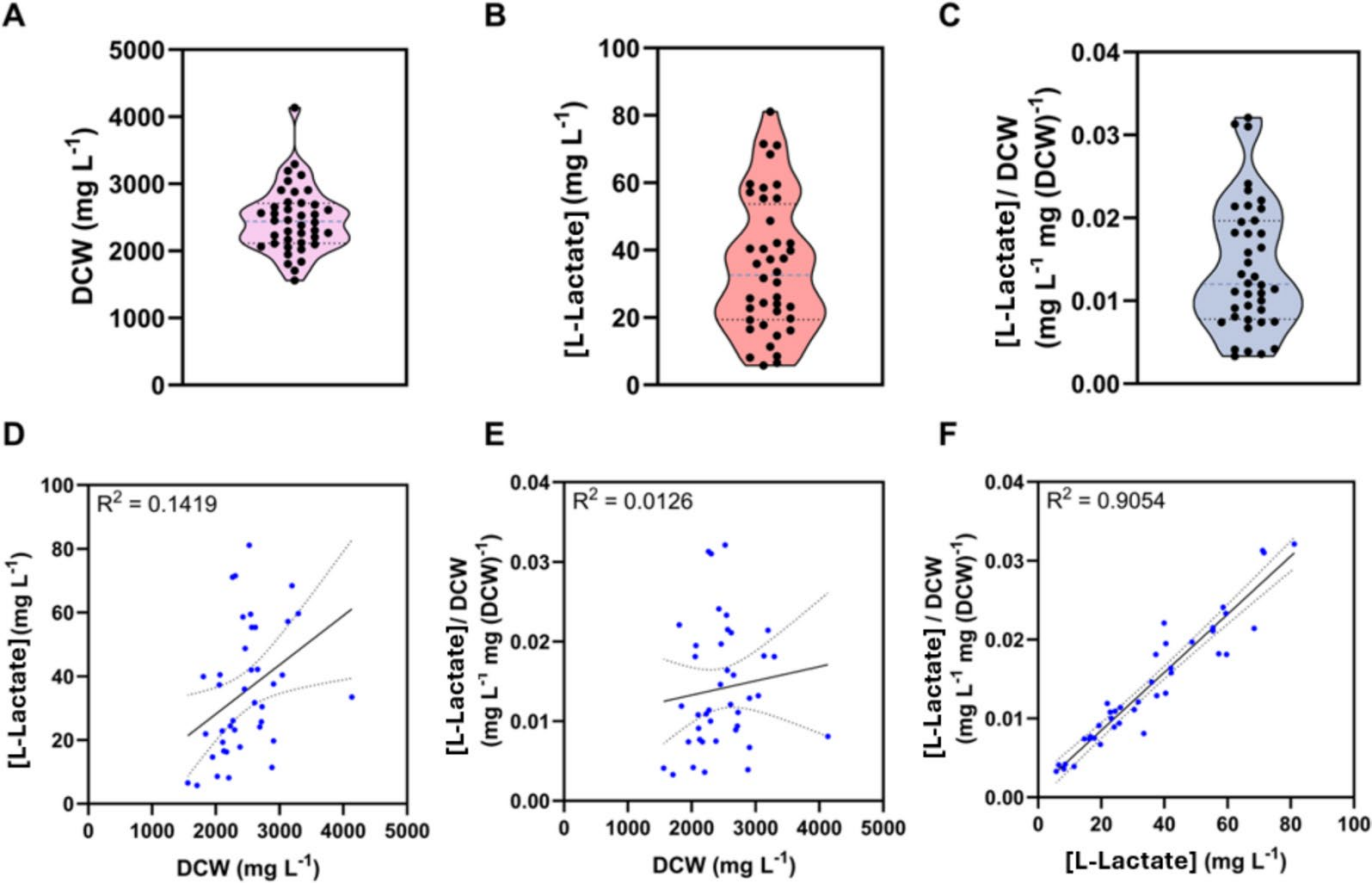
Performance metrics of the large full-factorial experiment. Conditions used for L-lactate production are specified in Supplementary Table 1 and the full data set in Supplementary Table 2. For the final time-point measurements, **A** a plot of dry cell weight, **B** a plot of L-lactate titre, **C** a plot of L-lactate yield (mg per mg of DCW), **D** an XY scatter plot of L-lactate titre against dry cell weight, **E** an XY scatter plot of L-lactate yield against dry cell weight, and **F** an XY scatter plot of L-lactate yield against L-lactate titre

With confidence instilled in the methodology, a larger DOE was performed to investigate 6 factors: light, HCO_3_^−^, NO_3_^−^, PO_4_^−^, glycerol, and light–dark cycle (Supplementary Table 1). A large full-factorial design allowed analysis of 1st, 2nd, and 3rd order interactions (Supplementary Table 1). This meant that interactions between up to 3 factors could be studied, revealing for example, that increasing light intensity, decreasing nitrogen, and using a light–dark cycle, could work synergistically to improve L-lactate productivity (Supplementary Fig. 10). Factor levels for phosphorus, bicarbonate, and glycerol were informed by previous experiments, while light intensity, nitrogen, and light–dark cycle values were influenced by a range of values described in the literature [9, 19, 20]. A growth phase was applied to increase biomass accumulation to an OD _720 nm_ of 2.0 before the media was changed to start a production phase. Cumulative biomass (as DCW) and L-lactate titre were measured after 4 days (Fig. 2), and a partial least square regression (PLSR) model was built for the output biomass accumulation, final L-lactate titre, and L-lactate yield (Supplementary Table 2). The PLSR model fit the data well with an R^2^ for predicted vs actual data of each output variable—biomass accumulation, L-lactate titre, and L-lactate yield—scoring highly at 0.97, 0.94, and 0.92, respectively (Supplementary Fig. 8). This suggests that the input factors described in the PLSR models can account for most of the variance of output.

The PLSR models reveal significant factors with the greatest weighting in determining output values. By sorting p-values, salient factors were extracted from the dataset to describe the conditions needed for optimisation. One of the most important factors for increased L-lactate titre and yield was the light–dark cycle (Supplementary Fig. 9. B and C). Interestingly, a light–dark cycle reduced the final biomass accumulation (Supplementary Fig. 9. A). The importance of a dark cycle for L-lactate titre is likely due to the change to fermentative metabolism where glycogen stores are consumed to provide reducing power for respiratory ATP synthesis. As oxygen is produced by photosynthesis during the day and retained in the flask, aerobic fermentation may occur during the dark period. In this process, glycogen undergoes saccharification and the resulting glucose is oxidised to CO_2_ by the OPPP, which produces NADPH, and by glycolysis and the TCA, which produces NADH [21]. Transcriptomic and fluxomic analysis reveals that during the dark period, the OPPP is preferentially upregulated compared to glycolysis, suggesting significant NADPH synthesis [22–24]. Because ldh_*Ll*_ preferentially consumes NADPH over NADH, the NADPH generation by the OPPP could be a key driver behind the observed L-lactate production if aerobic fermentation is co-occuring. A balance between NADH and NADPH is mediated by the pyridine nucleotide transhydrogenase (pntAB), which is essential for optimal growth rates under mixotrophic metabolism [25]. In the dark, NADH may be converted to NADPH by pntAB, providing the cofactor necessary for Ldh_*Ll*_ activity. However, previous reports suggest pntAB inactivity in dark conditions as a pntAB knockout gave no difference in autotrophic growth rate under light–dark conditions [25]. This suggests that the biggest driver of ldh_*Ll*_ activity could be NADPH evolved from the OPPP. Another hypothesis is that increased L-lactate synthesis may also occur due to a swelled pyruvate pool during the dark period. This does not align with a previous metabolomics study [26]. In the absence of oxygen, D-lactate, succinate, fumarate, αKG, and malate are produced by anaerobic fermentation [27]. To access the synthesis of these metabolites, flux must be routed through pyruvate in lower glycolysis, potentially providing a larger pyruvate pool for ldh_*Ll*_ to act on. The sealed flasks contained atmospheric air and would be subject to oxygen evolution from photosynthesis during the light periods. To access anaerobic fermentation, the oxygen content of the flask must first be exhausted by dark aerobic fermentation. It is not known what the oxygen content of the flasks was or how it varied throughout the time-course of the experiment. Gathering these data was beyond the scope of this study, but could be an interesting future direction. However, it appears likely that the consumption of glycogen and the evolution of CO_2_ decreased biomass and liberated stored carbon to be re-fixed and converted to L-lactate.

The second most important factor for L-lactate titre and yield was glycerol addition (Supplementary Fig. 9.B and C), which may be consumed to support mixotrophic growth, allowing for more carbon to be channelled to L-lactate production. Currently, there is no evidence for glycerol consumption by *Synechocystis* sp. PCC 6803.

Another important factor influencing L-lactate titre and yield was the interaction between nitrogen and bicarbonate concentrations (Supplementary Fig. 9). This two-factor interaction is visualised in Supplementary Figs. 10, 11, 12, and 13. These show that high HCO_3_^−^ and low nitrogen deliver the highest yields. This relationship is not strong enough to be incorporated into the predicted optimum factor levels for L-lactate titre and yield (Supplementary Fig. 11) instead, low nitrogen and low carbon are predicted to give the best results. Therefore, this two-factor interaction was not included in subsequent steps. On the contrary, the combination of a light–dark cycle with higher light intensity increased L-lactate yield (Supplementary Fig. 12), and both factor levels were included in the optimal conditions for titre and yield (Supplementary Fig. 9). Therefore, high light with the light–dark cycle was included for later experiments.

High CO_2_ supply can result in the secretion of extracellular carbohydrates, such as mannose [28–31]. The rationale behind including this factor was that L-lactate synthesis may replace the extracellular carbohydrate secretion as the carbon sink in this environment. The PSLR model showed that increased NaHCO_3_ does not improve titre (Supplementary Fig. 9.B and C). This is likely due to uncontrolled culture pH as 200 mM HEPES is insufficient to control the pH change of 160 mM NaHCO_3_ addition (Supplementary Fig. 3). The optimal pH for *Synechocystis* sp. PCC 6803 has previously been shown to be 7.5 [32], and deviation from this may be a physiological stressor that reduces carbon fixation and L-lactate synthesis.

Low phosphorus was included as it may slow DNA synthesis and growth whilst leaving L-lactate synthesis operational, thereby increasing the partitioning towards L-lactate synthesis. However, low amounts of phosphorus also reduced L-lactate titre and final DCW (Supplementary Fig. 9). This appears to show a shutting down of carbon fixation, potentially due to a global regulatory response that minimises metabolic activity in low-phosphorus environments.

**Fig. 3:**
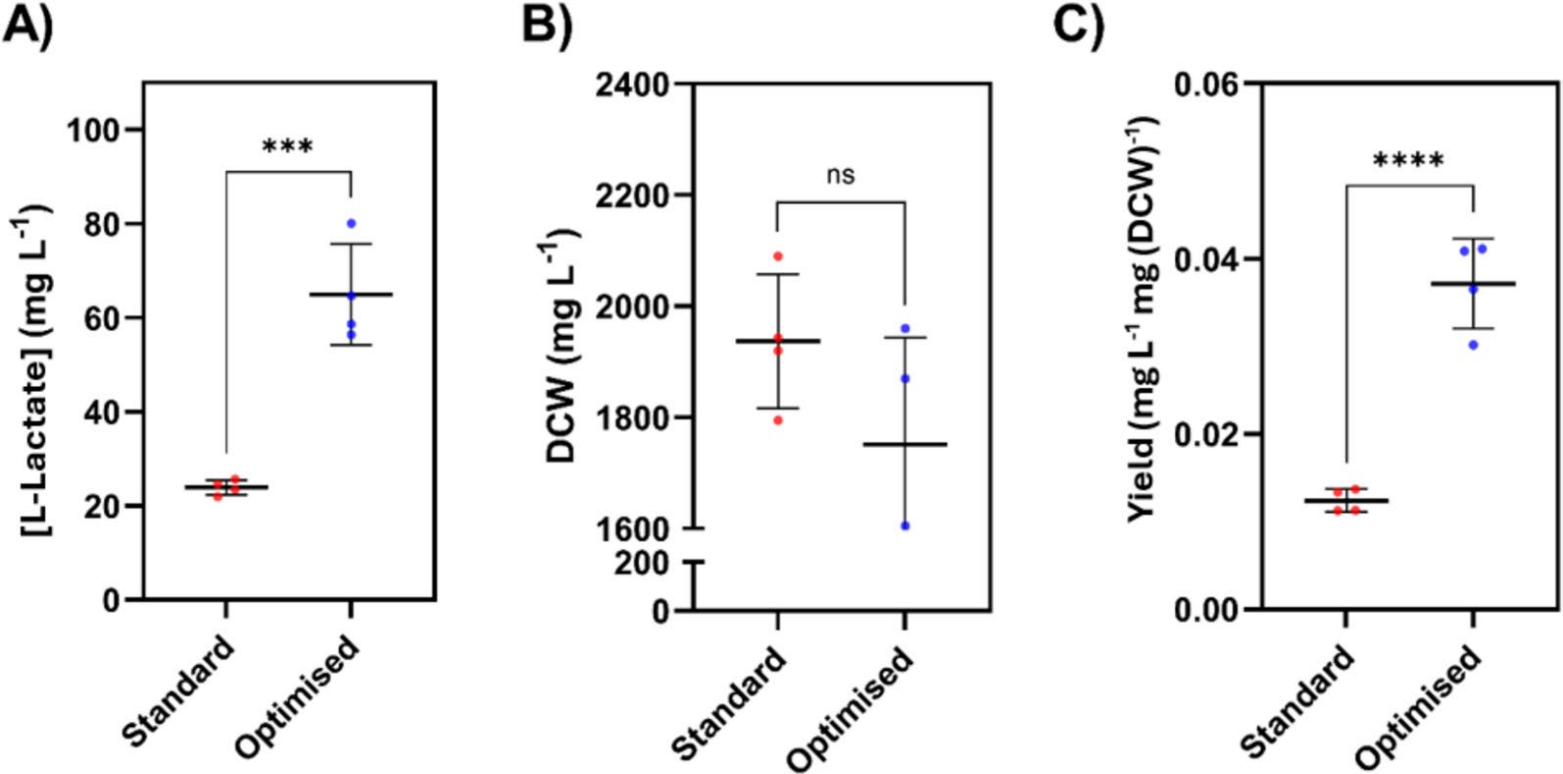
Validation of optimised conditions predicted by PLSR model. Values of output variables: **A** L-lactate titre, **B** biomass accumulation, and **C** L-lactate yield when optimised conditions are used. *** denotes *p* value < 0.001, **** denotes *p*-value < 0.0001, ns denotes no significant difference

To verify the model’s findings, the experiment was repeated, whereby quadruplet repeats of an optimised condition (16:8 light–dark cycle, 50 mM glycerol, 300 µmol (photons) m^−2^ s^−1^) were compared with a standard condition (constant illumination, no glycerol, 100 µmol (photons) m^−2^ s^−1^). Student’s *t*-test analysis resolved that the respective 2.6- and 3.0-fold increases in titre and yield were significant, corroborating the conclusions from the PLSRs. Demonstrated by the increase in L-lactate titre and yield, and decrease in final DCW, environmental factors appear to have the opposite effect on DCW, and total L-lactate titre and yield (Fig. 3). This suggests that dynamic control of media or conditions could be implemented to delineate growth and production phases. A two-stage approach could accrue biomass quickly and raise the TYR metrics of the biomass during the production phase. This idea has already been reviewed in the context of heterotrophic and autotrophic organisms [33, 34]. Publications advocating for autotrophic two-phase bio-production of L-lactate from *Synechocystis* sp. PCC 6803 utilise repression of the essential citrate synthase gene, resulting in growth arrest and L-lactate production [35, 36]. The genetic constructs involved in this method are unstable (presumably more so when scaled to industrial-sized bioreactors), and growth repression must be oscillated to prevent the accumulation of ATP [35].

### Optimisation of factors influencing L-lactate production

**Fig. 4:**
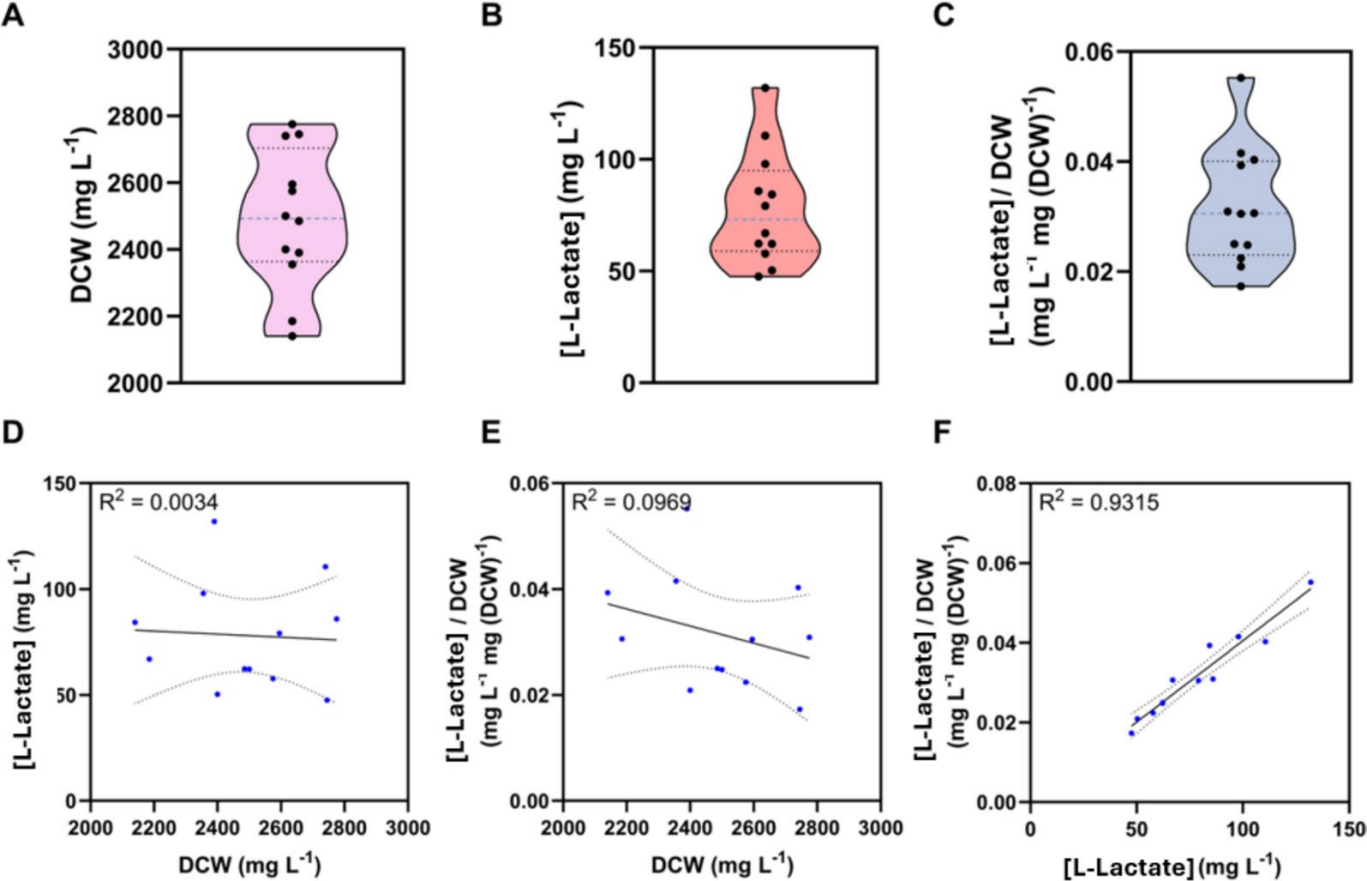
Performance metrics of the small full-factorial experiment. Conditions used for L-lactate production are specified in Supplementary Table 3. **A** a plot of dry cell weight, **B** a plot of L-lactate titre, **C** a plot of L-lactate yield (mg per mg of DCW), **D** an XY scatter plot of L-lactate titre against dry cell weight, **E** an XY scatter plot of L-lactate yield against dry cell weight, and **F** an XY scatter plot of L-lactate yield against L-lactate titre

Previously, DOE established a link between the light–dark cycle, glycerol addition, light intensity, and L-lactate production metrics. A new round of experimentation was designed to optimise these input factors for enhanced L-lactate titre and yield (Fig. 4). A new full-factorial design investigated the first, second, and third-order effects of the factors (Supplementary Table 3). After a 4-day growth and 4-day production phase, the data were used for building PLSR models. The PLSR model fit the data well with an R^2^ for predicted vs actual data of each output variable—biomass accumulation, L-lactate titre, and L-lactate yield—scoring highly at 0.85, 0.99, and 0.96, respectively (Supplementary Figs. 14). The lesser fit for biomass accumulation is understandable, as these factors were chosen to differentiate L-lactate, not biomass metrics.

**Fig. 5:**
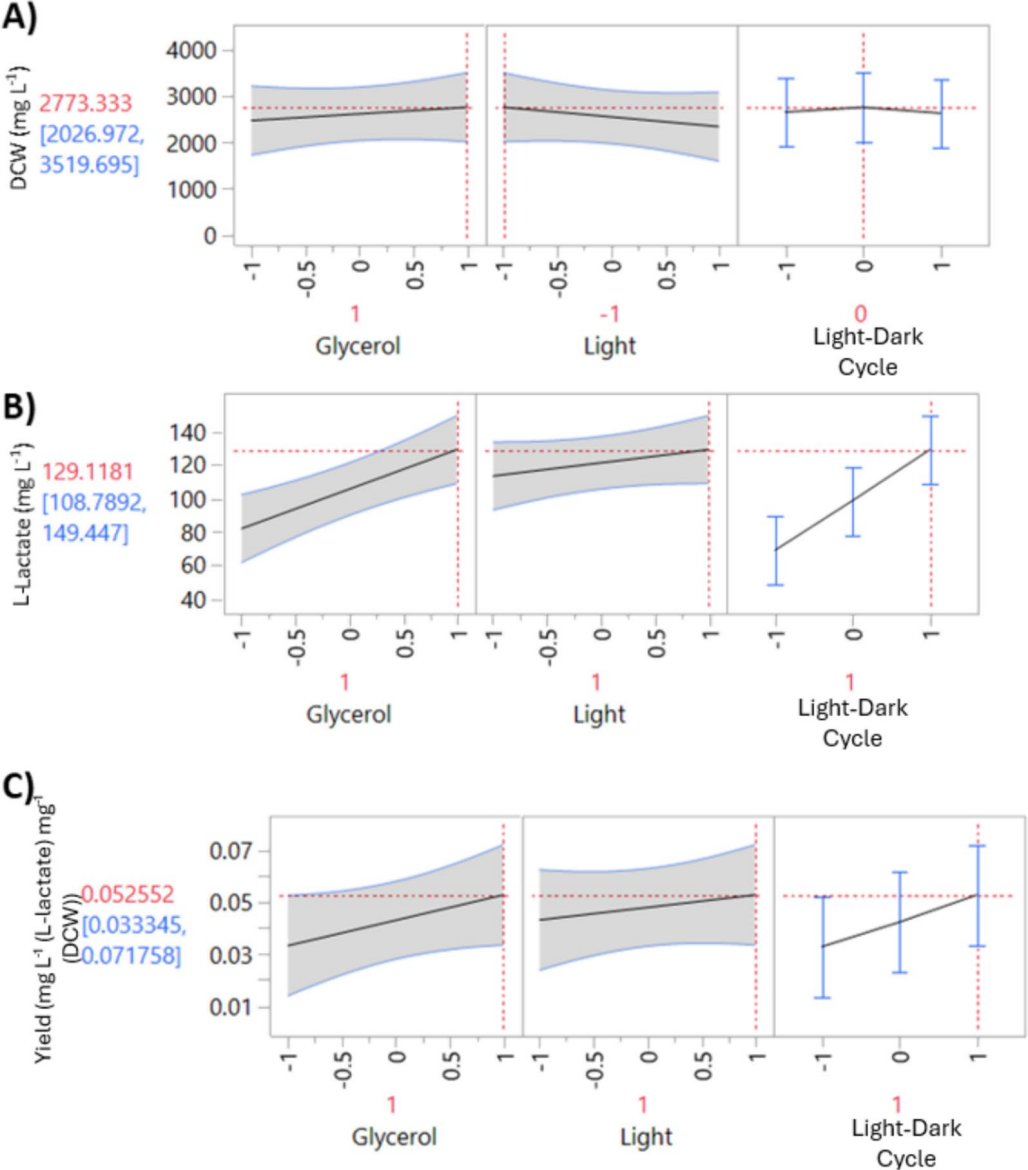
Modelling of input factor variables for optimisation of output factors. Models include **A** biomass accumulation,** B** L-lactate titre, and** C** L-lactate yield. Red crosses in graphs indicate the factor levels predicted to optimise the specified output variables. Y axis data: numbers in red are the output variables predicted when optimised conditions are used (red crosses). Darker shaded areas indicate 95% confidence intervals of line fitting

The PSLR models deemed that glycerol and light–dark cycle were the most significant factors for L-lactate metrics (Supplementary Fig. 15.B and C). In contrast to the previous DOE experiment, the second-order interaction between light intensity and light–dark cycle was significantly less significant (Supplementary Fig. 13.B and C and Supplementary Fig. 15.B and C). Indeed, light intensity did not appear to be a statistically significant factor, though there was a general trend of improved L-lactate titres and yield in higher light (Fig. 5 and Supplementary Fig. 15.B and C**)**. Similarly, no second-order interactions between factors delivered desirable results (Supplementary Fig. 15 and Supplementary Fig. 16). The best outcomes for titres and yield occur from longer dark periods, suggesting that yield in the dark period is where the maximum L-lactate production occurs (Fig. 5B, C). The other possibility is that during this dark period, cellular damage is repaired [22], metabolism is reorganised [23], and CO_2_ is released for maximum L-lactate production during the light period. Further work may elucidate the mechanism underlying the improvement.

A further increase in biomass accumulation, L-lactate titre and yield was seen at a higher glycerol content of 100 mM, suggesting that glycerol supplementation results in higher yield (Fig. 5.C). However, quantification of glycerol concentrations by HPLC before and after the production phase was performed and no convincing evidence was found for glycerol uptake (Supplementary Fig. 17). This may be because the resolution of HPLC is not enough to quantify any difference. No glycerol transporter has been identified in *Synechocystis* sp. PCC 6803, suggesting glycerol uptake and consumption are not necessarily the cause of increased titre. This finding is in line with a previous study, which found *Synechocystis* sp. PCC 6803 is unable to perform facultative photoheterotrophic growth in glycerol [37]. Whilst we cannot rule out the possibility of a non-specific membrane transporter that could import glycerol, future work may wish to express a glycerol transporter to study mixotrophy in *Synechocystis* sp. PCC 6803. Another hypothesis is that an environment high in molecules of osmotic potential, such as glycerol and salt, may cause a change in internal metabolism towards the synthesis of internal osmolytes. The main osmo-protectant, glucosylglycerol, is not directly associated with the glycolysis pathway. However, metabolomics has revealed that pyruvate pools are greater in BG11 media supplemented with 4% NaCl [38]. This excess pyruvate could support an increase in L-lactate synthesis. Another consideration is that L-lactate synthesis may be a useful internal osmolyte, and synthesis may balance osmotic potential between the cell’s internal and external environment. Experiments resolving this question were outside the scope of this study; however, we hypothesise that the osmotic properties of internally synthesised L-lactate may be advantageous in a high glycerol environment and could drive increased L-lactate synthesis through metabolic rewiring.

The outcome of this experiment was a new set of optimised conditions where high glycerol (100 mM), long dark periods (12:12 h), and high light (300 µmol (photons) m^−2^ s^−1^ increase L-lactate production metrics (Fig. 4 and Fig. 5).

### Validation of optimised condition

To validate that a new set of optimal conditions had been found for L-lactate production, a validation experiment was performed. Four groups were used, including standard BG11, the worst conditions for L-lactate metrics, and the best with and without optimised nitrogen and phosphorus levels (Supplementary Table 5). Quintuplet repeats of each condition were incubated for a 4-day growth, diluted to an OD _720 nm_ of 2.0, and incubated for a 4-day production phase. L-lactate titre, DCW, and optical density were measured at the end of the experiment and analysed by one-way ANOVA.

Mean L-lactate titres recorded for the worst, normal, optimised (−N), and optimised (+ N) were 23.8, 20.1, 90.5, and 126.6 mg L^−1^, respectively (Supplementary Fig. 18). The difference between the worst and normal BG11 conditions was not significant; however, all other comparisons revealed a significant difference (p < 0.0005). The lack of difference between the worst and standard BG11 conditions suggests that 1.15 µM (5%) K_2_HPO_4_ is insufficient to affect outputs. Whilst the factor level was previously set to 0 µM, this appeared to halt growth and L-lactate production (Supplementary Fig. 7). Therefore, phosphorus limitation does not appear to be a useful direction for enhanced L-lactate yield or a two-stage production set-up.

The initial screening experiment suggested that nitrogen limitation was needed for optimal L-lactate titre and yield (Supplementary Table 2 and Supplementary Fig. 9), but it was considered not to be a significant factor (Supplementary Fig. 13). Therefore, optimal conditions (100 mM glycerol, and 12: 12 h light: dark) in normal (+ N) and low nitrogen (−N) were tested. L-Lactate titres were significantly higher in + N conditions, suggesting that nitrogen limitation was not conducive to maximising the final titre. The bounds for the supplementation of glycerol were set by a separate experiment (Supplementary Fig. 19).

Yield was similarly affected, with the only non-significant difference between worst and standard BG11 conditions (*p* < 0.05, Supplementary Fig. 18). A 7.4- and 5.2-fold increase in yield was recorded from standard BG11 to the optimised + N and − N conditions, respectively (Supplementary Fig. 18.D). Biomass accumulation, measured by DCW, revealed that nitrogen limitation had not significantly limited growth (Supplementary Fig. 18.C), although this was not reflected in OD _720 nm_ data, which showed a significant impingement on growth (Supplementary Fig. 18.B). The difference could be attributed to changes in cell shape, size, and composition under nitrogen limitation. This was not however, apparent from any two-factor interaction, and may be due to a higher-order interaction (Supplementary Fig. 10).

**Fig. 6:**
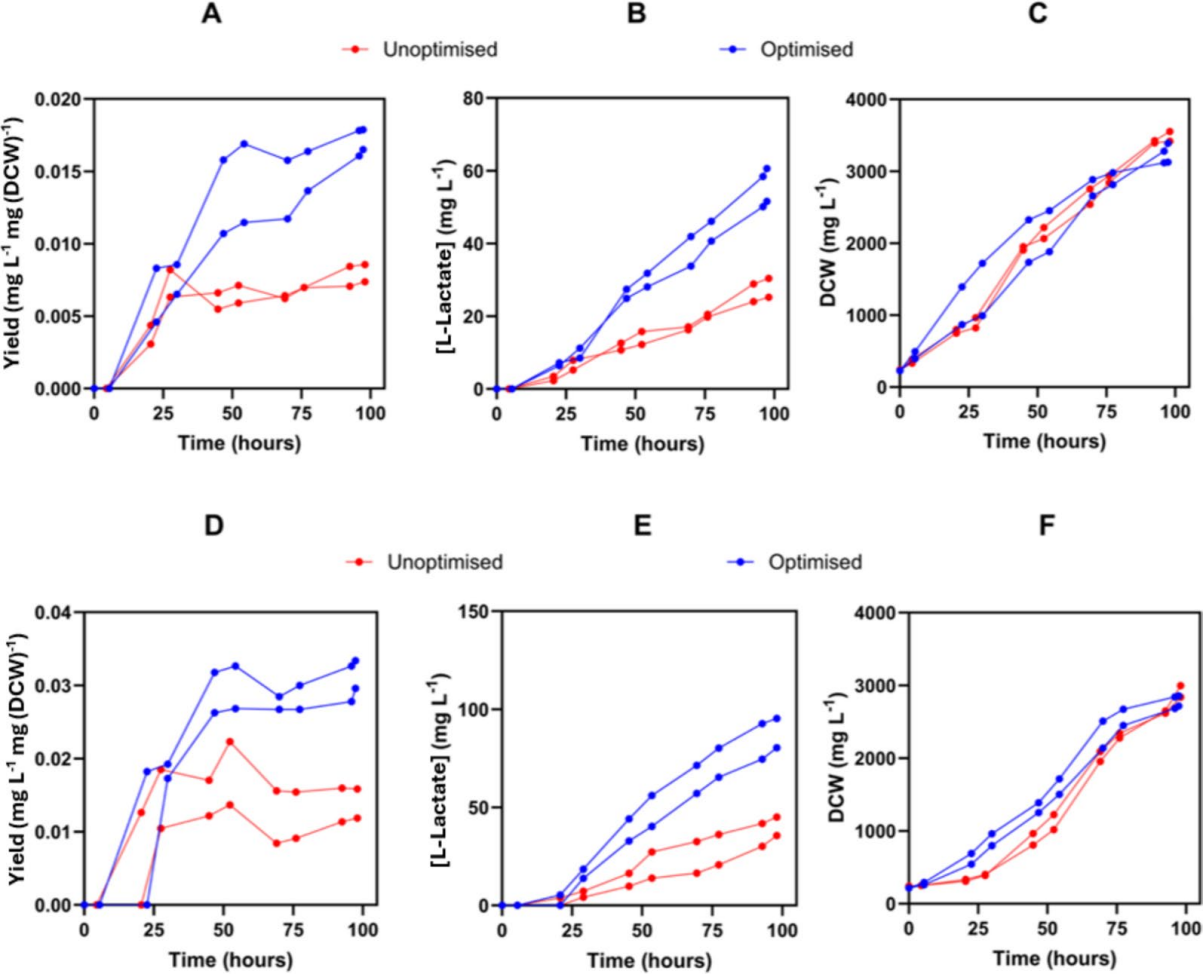
Growth and L-lactate production at 2 L scale for optimised and unoptimised conditions. **A** L-lactate yield vs time, **B** L-lactate titre vs time, and **C** DCW vs time plots of four photobioreactors, duplicate optimised photobioreactors 1 and 2, and unoptimised photobioreactors 3 and 4. **D** L-lactate yield vs time, **E** L-lactate titre vs time, and F DCW vs time plots of four closed flasks, duplicate optimised flasks 1 and 2, and unoptimised flasks 3 and 4

To test the scalability of these optimum L-lactate production conditions determined by the iterative DOE process, an open (Fig. 6A, B and C) and closed (Fig. 6D, E and F) 2-L system for L-lactate was run in duplicate under the optimised (reactors 1 & 2) and unoptimised (reactors 3 & 4) conditions (Supplementary Fig. 21). In the open system, a 2-L CSTR (classic stirred tank reactor), the optimised conditions did not improve L-lactate titre as markedly as in the closed system, a sealed 5-L flask containing 2-L culture. The optimised conditions improved L-lactate titres and yield in both open and closed systems, and the closed system was remarkably consistent with the DOE (Fig. 6). The closed system in flasks had higher L-lactate titre, yield, and rate but lower growth than the open system in PBR’s (Supplementary Table 6). This perhaps suggests a two-phase process whereby a PBR-like open system is used for the accumulation of biomass, leveraging the faster growth rates and a flask-like closed system is used for production, leveraging the higher yield.

These results solidify the findings of the preceding DOE experiments as the optimised conditions of the significant factors gave the best results. Following two rounds of optimisation, a 6.3- and 7.4-fold increase in titre and yield were observed at 50 ml scale, a 2.1- and 2.2-fold increase in 2-L photobioreactors and a 2.3- and 2.4-fold increase in 2-L closed flasks. Combining this process with genetic engineering could further improve titres, for example, better performing ldh_*Ll*_ constructs are available [39] and metabolic engineering of glycerol importers has been achieved in *Synechococcus* PCC 7942 [40]. Parallel DOE optimisations could also be done on strains modified for glycogen and PHB knockouts or modifications in the photosynthetic electron transport chain, revealing if and how optimal conditions change between chassis. Since DOE experiments optimise specifically for the conditions which were used, future work may explore the use of light–dark cycles, high light, and glycerol addition to judge if the benefits extend to other large-scale cultivation systems.

## Conclusions

There is a need to find new methods to improve bio-product titres from *Synechocystis* sp. PCC 6803. In this work, DOE methodology is used iteratively to explore the prospect of engineering environmental factors for increased production and to delineate complex multivariate interactions between factors in an efficient manner. A subset of factors was identified and optimised (light–dark cycle, glycerol, light intensity), resulting in a 6.3- and 7.4-fold improvement in L-lactate titre and yield, respectively. These optimised conditions could be implemented in other projects to improve yield metrics. Alternatively, as the work verified the accuracy of DOE methodology, the process may be repeated to identify new factors that significantly improve the titres of other bio-products. This work may be expanded upon by testing highly modified bio-production chassis. For example, strains deficient in glycogen and PHB synthesis could be tested and may interact differently with the environmental conditions tested here. Further to its utility as an adjunct tool in metabolic engineering, this work may be developed to build a two-phase bioreactor strategy by the bioprocess engineering community. This may push the economic feasibility of cyanobacterial biotechnology in the right direction towards commercialisation.

## Materials and methods

An engineered strain of *Synechocystis* sp PCC 6803 (similar to SAA023 [41]) was used, containing a plasmid (Supplementary Fig. 22), encoding the *Lactococcus lactis L-ldh1* gene with an L39R substitution [35, 39]. This strain was confirmed to produce L-lactate photosynthetically from CO_2_ by assay (Megazyme D-/L-Lactic acid kit) and HPLC. L-Lactate yield was optimised using design of experiments and predictive modelling in JMP (JMP Pro 16 from SAS Institute, a statistical discovery program) in a similar way to a previous study [7] based on the principles outlined in a recent review [4].

### Standard closed culturing conditions for *Synechocystis* sp. PCC 6803

In all experiments, flasks were incubated at 34 °C, 120 rpm, in an Infors HT Multitron incubator with a modified shaking platform that provided illumination from the bottom (Supplementary Fig. 5). Illumination was provided by warm white LEDs (TruOpto OSFY016M510), and the intensity was set using a photometer (LI-COR biosciences, LI-250A). Unless otherwise specified, all closed flask cultures used standard BG11 media, which was modified by adding 200 mM HEPES buffer (adjusted to pH 7.2 with 10 M KOH) and 80 mM NaHCO_3_. Erlenmeyer flasks were sealed by fastening 2 layers of Sarolgold film over the receptacle opening with 2 elastic bands (Supplementary Fig. 1).

### Construction of the L-lactate-producing strain

As a kind gift from Dr Elton P. Hudson, a plasmid (SAA023) was obtained, which encoded the *L-ldh1* gene (with L39R substitution from *Lactococcus lactis,* which increases proclivity to NADPH over NADH) and chloramphenicol resistance inside homology arms, which would mediate genome insertion at the genome locus *slr0168* [35]. Upon receipt, whole plasmid sequencing was performed using Oxford Nanopore long-read technology facilitated by Plasmidsaurus, which confirmed the integrity of the plasmid (Supplementary Fig. 22). The plasmid was transformed into *Synechocystis* by natural transformation and colonies were screened for integration of the plasmid by PCR with bespoke primers (Supplementary Fig. 23).

### Transformation of *Synechocystis* sp. PCC 6803

Initially, 10 mL of BG11 was inoculated to OD _720 nm_ with the desired background strain for transformation. The culture was incubated under 30 µmol (photons) m^−2^ s^−1^, 32° C, and 120 rpm until an OD _720 nm_ of 1.0–2.0 had been achieved. 10 mL of culture was transferred to a 15-mL tube (Sarstedt) and centrifuged (Eppendorf Centrifuge 5810 R, at 25 °C, 4000 rpm, rotor A-4-62) for 10 min until the cells had pelleted. The supernatant was discarded, and the cells were re-suspended in 5 mL of fresh BG11 before another round of centrifugation (Eppendorf Centrifuge 5810 R, at 25 °C, 4000 rpm, rotor A-4-62) was performed for 10 min to pellet the cells. The supernatant was discarded and the cells were re-suspended in 200 µL of fresh BG11. From a previous plasmid purification (Omega Biotek plasmid DNA mini kit 1, used as per the manufacturer’s instructions), 2 µg of the desired plasmid was added to the 200 µL culture, and the tube was gently flicked 5–10 times to mix the contents. The 15-mL tube (Sarstedt) containing the culture and plasmid was incubated in an Algaetron AG130 at 30 µmol (photons) m^−2^ s^−1^ and 32 °C for 6 h. The 200 µL culture was then spread onto a non-selective BG11 agar plate and left to dry in a laminar flow hood for 1 h. The dried plate was incubated in an Algaetron AG130 at 15 µmol (photons) m^−2^ s^−1^, 32 °C for 24 h. After 24 h, 500 µL of liquid BG11 was used to re-suspend the cells and transfer them to a BG11 plate containing antibiotics. The plate was allowed to dry in a laminar flow hood for 1 h, and the resulting plate was wrapped in parafilm (Ancor) and incubated at 30 µmol (photons) m^−2^ s^−1^, 32 °C, for 1 month. Once colonies were visible, they were streaked over a larger patch on a new BG11 plate containing antibiotics and incubated at 30 µmol (photons) m^−2^ s^−1^, 32 °C, for 1 week. Growth on the patch was used to inoculate a 5 mL BG11 culture in a 25-mL flask (Erlenmeyer), which was incubated in an Infors HT Multitron incubator under 30 µmol (photons) m^−2^ s^−1^, at standard conditions. Once the culture OD _720 nm_ reached 3.0–5.0, 4 mL of culture was transferred to a 15-mL tube (Sarstedt) and centrifuged (Eppendorf Centrifuge 5810 R, at 25 °C, 4000 rpm, rotor A-4-62) for 10 min until the cells had pelleted and were re-suspended in 1 mL of BG11 with 8% (v / v) dimethyl sulfoxide (DMSO). The 1-mL culture was transferred to a cryotube (Sarstedt CryoPure), frozen in liquid nitrogen, and stored in a -80 °C freezer (New Brunswick Scientific).

### Detection of L-lactate by enzyme assay kit

To measure L-lactate production, 1 ml of cell culture was transferred to a 1.5-mL tube (Sarstedt) and centrifuged (Fisher Scientific accuSpin Micro, 1300 rpm, 2 min) to pellet the cells. The supernatant was transferred to a mini-spin column (Protein Ark Proteus Mini Clarification Spin Column) and centrifuged (Fisher Scientific accuSpin Micro, 1300 rpm, 2 min). The clarified flow-through was then stored at -20 °C (< 1 month) until required for use in the L-lactate assay. To ascertain the L-lactate content of a sample, 1 mL of clarified supernatant was harvested from a culture and used in a 100 µL reaction mixture using the L-lactate kit as per the manufacturer’s instructions (Megazyme D-/L-Lactic acid kit). The absorbance assay was performed in a 96-well plate (Greiner 96-well microplate F-bottom) in a plate reader (BMG Labtech FLUOstar Omega). Before L-lactate dehydrogenase addition, an absorbance scan was performed (25 °C incubation, > 30 s double orbital shaking at 600 rpm between scans, average well scanning used, 5 time-points taken over > 5 min). After L-lactate dehydrogenase addition, an absorbance scan was performed (25 °C incubation, > 30 s double orbital shaking at 600 rpm between scans, average well scanning used, regular time-points taken over > 30 min) and a time-point selected where increases in absorbance of all wells had ceased. Standard curves were performed on each run and L-lactate concentrations were calculated as per the manufacturer’s instructions.

### Design of experiments—experimental L-lactate optimisation

DOE experiments were performed according to a bespoke experiment matrix (Supplementary Table 1 and Supplementary Table 3). Initially, a 2-mL sample of the L-lactate-producing strain SAA023 was recovered from a − 80 °C stock and used to inoculate a 10 mL liquid BG11 culture. This 12 mL culture was grown in a 25 mL flask (Erlenmeyer) under low light (30 µmol (photons) m^−2^ s^−1^) in standard conditions for 3 days to an OD _720 nm_ between 2.0 and 4.0. The culture was then used to inoculate BG11 media in a new set of sealed 250 mL flasks (Erlenmeyer) to a final volume of 50 mL at OD _720 nm_ 0.1. To conclude the growth stage, the 50 mL cultures were incubated under standard conditions at 100 µmol (photons) m^−2^ s^−1^ for 4 days, to achieve an OD _720 nm_ between 4.0 and 6.0. To initiate the production phase, OD _720_ measurements were taken for each flask to calculate the volume needed to inoculate a 25 mL culture to an OD _720 nm_ of 2.0. The appropriate volume of culture was transferred to a 15 mL tube (Sarstedt) and centrifuged (Eppendorf Centrifuge 5810 R, at 25 °C, 4000 rpm, rotor A-4-62) until the cells had pelleted. The supernatant was removed and the cells re-suspended in a unique media corresponding to a condition in the DOE matrix. To make this media, glycerol (Fisher Scientific 99% analytical reagent grade) was added from an autoclaved 50% (v/v) in water stock. Each condition was contained in a sealed 100 mL flask (Erlenmeyer) with 25 mL of total culture volume at an OD _720 nm_ of 2.0. At this point, OD _720 nm_ and L-lactate measurements were taken. Cultures were incubated under standard conditions but with the necessary environmental factor changes implemented for each flask. Light–dark cycles were performed by covering flasks with a black cloth in a separate incubator without light. After 4 days, the experiment was concluded with L-lactate, optical density, and DCW measurements. Some conditions induced flocculation, leaving DCW measurements as the best option for measuring culture density; accordingly, the culture was processed for cell dry weight measurement. A standard curve was constructed to correlate OD to DCW (Supplementary Fig. 20). Chloramphenicol was added at the start of each culture stage to a final concentration of 10 ng mL^−1^.

### Design of experiments—design and analysis

All DOE experiments were designed and analysed using JMP Pro 16 (SAS Institute). Different DOE design matrices were used based on varying needs and objectives. The custom design tool was used to build bespoke experiments where some or all 1st, 2nd, or 3rd order interactions were tested for. 1st order interactions (main effects): effect on the output variable from a single input factor; 2nd order interactions (two-factor interactions): effect on the output variable from interactions between two input factors; and 3rd order interactions (three-factor interactions): effect on the output variable from interactions between three input variables.

The large full-factorial experiment (Supplementary Table 1) was performed to find whether the input variables light (100, 300 µmol (photons) m^−2^ s^−1^), NaNO_3_ (0.88, 8.8, 17.6 mM), K_2_HPO_4_ (1.15, 11.5, 23 µM), NaHCO_3_ (40, 60, 80 mM), glycerol (0, 25, 50 mM), and light–dark cycle (24:0, 16:8 light:dark) have a significant impact on the output variables DCW (g L^−1^), L-lactate titre (mg L^−1^), and L-lactate titre per DCW (mg (L-lactate) g^−1^ (DCW) L^−1^). All permutations of 1st, 2nd, and 3rd order interactions were included in the design, as well as a midpoint. Factors were deemed significant when p < 0.01.

The small full-factorial experiment (Supplementary Table 300) was performed to find whether the input variables light (100, 300 µmol (photons) m^−2^ s^−1^), glycerol (50,100 mM), and light–dark cycle (20:4, 16:8,12:12 light:dark) have a significant impact on the output variables DCW (g L^−1^), L-lactate titre (mg L^−1^), and L-lactate titre per DCW (mg (L-lactate) g^−1^ (DCW) L^−1^). All permutations of 1st, 2nd, and 3rd order interactions were included in the design. Factors were deemed significant when *p* < 0.05.

### Quantification of glycerol uptake

Glycerol uptake was quantified by HPLC. 1 ml of cell culture was transferred to a 1.5 ml tube (Sarstedt) and centrifuged (Fisher Scientific accuSpin Micro, 1300 rpm, 2 min) to pellet the cells. The supernatant was transferred to a mini-spin column (Protein Ark Proteus Mini Clarification Spin Column) and centrifuged (Fisher Scientific accuSpin Micro, 1300 rpm, 2 min). The clarified flow-through was analysed by an Agilent 1260 Infinity HPLC with a 1260 ALS auto-sampler. 10 μL injections were analysed isocratically on an Agilent Hi-Plex H column (300 × 7.7 mm; 2.5 mM H_2_SO_4_) at 60 °C with a flow rate of 0.7 mL min^−1^ for 27 min and detected by RID. Glycerol was quantified by peak area against a standard curve of known concentrations of analytical quality glycerol standard (> 99%, Sigma). Representative HPLC traces can be found in Supplementary Fig. 17.

### Scale up from flask to 2-L reactor or 5-L flask

The growth stage was conducted for batch scale up to the 50 mL cultures, as described above. These were used to make 200 mL cultures in 500-mL sealed flasks at OD 720 nm 2.0 grown for 4 days to reach an OD 720 nm of ~ 7.0–8.0. To initiate the 2-L CSTR or 5-L flask production phase, OD _720_ measurements were taken for each flask to calculate the volume needed to inoculate a 2-L culture to an OD _720 nm_ of 2.0. This was transferred to a 2-L Applikon Bio vessel (https://www.getinge.com/int/products/applikon-bio/) attached to a Getinge LivitFlex control unit (https://www.getinge.com/int/products/livit-flex/). We lit the vessels with warm white light delivered by LED strips (ZFS-8500HDWW, JKL UK) attached directly to the reactor; the length of the LED strip used determined light intensity (Supplementary Fig. 21). The power supply to the LED’s was on a timer to facilitate the light–dark cycle. Light intensity per cm of LED strip added was measured by a light meter in the centre of the vessel when empty and used to calculate the correct intensity. The 5 L flasks were prepared and closed in the same way as the flasks for the DOE. These were lit by a large, fixed intensity, warm white, LED spot lamp (Supplementary Fig. 21), and light intensity was controlled by the distance of the flask from the light source. The light intensity was measured by a light meter in the centre of the empty flask.

## Supplementary Information

Supplementary Material 1.

## Data Availability

No datasets were generated or analysed during the current study.

## References

[1] Al-Haj L, Lui YT, Abed RMM, Gomaa MA, Purton S. Cyanobacteria as chassis for industrial biotechnology: progress and prospects. Life. 2016;6:42. 27916886 10.3390/life6040042 PMC5198077

[2] Cheng J, Zhang K, Hou Y. The current situations and limitations of genetic engineering in cyanobacteria: a mini review. Mol Biol Rep. 2023;50:5481–7. 37119415 10.1007/s11033-023-08456-8

[3] Srivastava A, Summers ML, Sobotka R. Cyanobacterial sigma factors: current and future applications for biotechnological advances. Biotechnol Adv. 2020;40:107517. 31945415 10.1016/j.biotechadv.2020.107517

[4] Andrews F, Faulkner M, Toogood HS, Scrutton NS. Combinatorial use of environmental stresses and genetic engineering to increase ethanol titres in cyanobacteria. Biotechnol Biofuels. 2021;14:240. 34920731 10.1186/s13068-021-02091-w PMC8684110

[5] Hauf W, et al. Metabolic changes in *Synechocystis* PCC6803 upon nitrogen-starvation: excess NADPH sustains polyhydroxybutyrate accumulation. Metabolites. 2013;3:101–18. 24957892 10.3390/metabo3010101 PMC3901256

[6] Nakajima T, Yoshikawa K, Toya Y, Matsuda F, Shimizu H. Metabolic flux analysis of the *Synechocystis* sp. PCC 6803 ΔnrtABCD mutant reveals a mechanism for metabolic adaptation to nitrogen-limited conditions. Plant Cell Physiol. 2017;58:537–45. 28130420 10.1093/pcp/pcw233

[7] Faulkner M, Andrews F, Scrutton N. Improving productivity of citramalate from CO2 by *Synechocystis* sp. PCC 6803 through design of experiment. Biotechnol Biofuels Bioprod. 2024;17:143. 39639409 10.1186/s13068-024-02589-z PMC11622482

[8] Canonico M, Konert G, Kaňa R. Plasticity of cyanobacterial thylakoid microdomains under variable light conditions. Front Plant Sci. 2020;11:586543. 33304364 10.3389/fpls.2020.586543 PMC7693714

[9] Du W, et al. Exploiting day- and night-time metabolism of *Synechocystis* sp. PCC 6803 for fitness-coupled fumarate production around the clock. ACS Synth Biol. 2019;8:2263–9. 31553573 10.1021/acssynbio.9b00289 PMC6804261

[10] Barone GD, et al. Towards the rate limit of heterologous biotechnological reactions in recombinant cyanobacteria. Biotechnol Biofuels Bioprod. 2023;16:4. 36609316 10.1186/s13068-022-02237-4 PMC9825001

[11] Cano M, et al. Glycogen synthesis and metabolite overflow contribute to energy balancing in cyanobacteria. Cell Rep. 2018;23:667–72. 29669272 10.1016/j.celrep.2018.03.083

[12] Le Riche R, Picheny V. Revisiting Bayesian optimization in the light of the COCO benchmark. Struct Multidiscip Optim. 2021;64:3063–87.

[13] Mockus J. Global optimization and the bayesian approach. In: Mockus J, editor. Bayesian approach to global optimization theory and applications. Dordrecht: Springer; 1989.

[14] Balakrishnan R, Mohan N, Sivaprakasam S. Chapter 11 - Application of design of experiments in bioprocessing: process analysis, optimization, and reliability. In: Sirohi R, Pandey A, Taherzadeh MJ, Larroche C, editors. Current developments in biotechnology and bioengineering. Amsterdam: Elsevier; 2022.

[15] Keskin Gündoğdu T, Deniz İ, Çalışkan G, Şahin ES, Azbar N. Experimental design methods for bioengineering applications. Crit Rev Biotechnol. 2016;36:368–88. 25373790 10.3109/07388551.2014.973014

[16] Yadav I, et al. Enhancement of isoprene production in engineered *Synechococcus elongatus* UTEX 2973 by metabolic pathway inhibition and machine learning-based optimization strategy. Bioresour Technol. 2023;387:129677. 37579861 10.1016/j.biortech.2023.129677

[17] Ranakoti L, et al. Critical review on polylactic acid: properties, structure, processing, biocomposites, and nanocomposites. Materials. 2022;15:4312. 35744371 10.3390/ma15124312 PMC9228835

[18] Żymańczyk-Duda E, Samson SO, Brzezińska-Rodak M, Klimek-Ochab M. Versatile applications of cyanobacteria in biotechnology. Microorganisms. 2022;10:2318. 36557571 10.3390/microorganisms10122318 PMC9785398

[19] Gründel M, Scheunemann R, Lockau W, Zilliges Y. Impaired glycogen synthesis causes metabolic overflow reactions and affects stress responses in the cyanobacterium *Synechocystis* sp. PCC 6803. Microbiology. 2012;158:3032–43. 23038809 10.1099/mic.0.062950-0

[20] Namakoshi K, Nakajima T, Yoshikawa K, Toya Y, Shimizu H. Combinatorial deletions of *glgC* and *phaCE* enhance ethanol production in *Synechocystis* sp. PCC 6803. J Biotechnol. 2016;239:13–9. 27693092 10.1016/j.jbiotec.2016.09.016

[21] Yang C, Hua Q, Shimizu K. Metabolic flux analysis in *Synechocystis* using isotope distribution from ^13^C-labeled glucose. Metab Eng. 2002;4:202–16. 12616690 10.1006/mben.2002.0226

[22] Diamond S, et al. Redox crisis underlies conditional light-dark lethality in cyanobacterial mutants that lack the circadian regulator, RpaA. Proc Natl Acad Sci USA. 2017;114:E580–9. 28074036 10.1073/pnas.1613078114 PMC5278464

[23] Diamond S, Jun D, Rubin BE, Golden SS. The circadian oscillator in *Synechococcus elongatus* controls metabolite partitioning during diurnal growth. Proc Natl Acad Sci U S A. 2015;112:E1916–25. 25825710 10.1073/pnas.1504576112 PMC4403147

[24] Kugler A, Stensjö K. Optimal energy and redox metabolism in the cyanobacterium *Synechocystis* sp. PCC 6803. npj Syst Biol Appl. 2023;9:1–13. 37739963 10.1038/s41540-023-00307-3 PMC10516873

[25] Kämäräinen J, et al. Pyridine nucleotide transhydrogenase PntAB is essential for optimal growth and photosynthetic integrity under low-light mixotrophic conditions in *Synechocystis* sp. PCC 6803. New Phytol. 2017;214:194–204. 27930818 10.1111/nph.14353

[26] Will SE, et al. Day and night: metabolic profiles and evolutionary relationships of six axenic non-marine cyanobacteria. Genome Biol Evol. 2019;11:270–94. 30590650 10.1093/gbe/evy275 PMC6349668

[27] Hasunuma T, Matsuda M, Kondo A. Improved sugar-free succinate production by *Synechocystis* sp. PCC 6803 following identification of the limiting steps in glycogen catabolism. Metab Eng Commun. 2016;3:130–41. 29468119 10.1016/j.meteno.2016.04.003 PMC5779724

[28] De Philippis R, Sili C, Vincenzini M. Response of an exopolysaccharide-producing heterocystous cyanobacterium to changes in metabolic carbon flux. J Appl Phycol. 1996;8:275–81.

[29] Gupta JK, Rai P, Jain KK, Srivastava S. Overexpression of bicarbonate transporters in the marine cyanobacterium *Synechococcus* sp. PCC 7002 increases growth rate and glycogen accumulation. Biotechnol Biofuels. 2020;13:17. 32015756 10.1186/s13068-020-1656-8 PMC6988372

[30] Kamennaya NA, et al. Installing extra bicarbonate transporters in the cyanobacterium *Synechocystis* sp. PCC6803 enhances biomass production. Metab Eng. 2015;29:76–85. 25769289 10.1016/j.ymben.2015.03.002

[31] Stöckel J, Elvitigala TR, Liberton M, Pakrasi HB. Carbon availability affects diurnally controlled processes and cell morphology of *Cyanothece* 51142. PLoS ONE. 2013;8:e56887. 23457634 10.1371/journal.pone.0056887 PMC3574086

[32] Touloupakis E, Cicchi B, Benavides AMS, Torzillo G. Effect of high pH on growth of *Synechocystis* sp. PCC 6803 cultures and their contamination by golden algae (*Poterioochromonas* sp.). Appl Microbiol Biotechnol. 2016;100:1333–41. 26541331 10.1007/s00253-015-7024-0 PMC4717179

[33] Burg JM, et al. Large-scale bioprocess competitiveness: the potential of dynamic metabolic control in two-stage fermentations. Curr Opin Chem Eng. 2016;14:121–36.

[34] Shabestary K, et al. Design of microbial catalysts for two-stage processes. Nat Rev Bioeng. 2024. 10.1038/s44222-024-00225-x.

[35] Shabestary K, et al. Cycling between growth and production phases increases cyanobacteria bioproduction of lactate. Metab Eng. 2021;68:131–41. 34601120 10.1016/j.ymben.2021.09.010

[36] Shabestary K, et al. Targeted repression of essential genes to arrest growth and increase carbon partitioning and biofuel titers in cyanobacteria. ACS Synth Biol. 2018;7:1669–75. 29874914 10.1021/acssynbio.8b00056

[37] Rippka R, Deruelles J, Waterbury JB, Herdman M, Stanier RY. Generic assignments, strain histories and properties of pure cultures of cyanobacteria. Microbiology. 1979;111:1–61.

[38] Klähn S, et al. Integrative analysis of the salt stress response in cyanobacteria. Biol Direct. 2021;16:26. 34906211 10.1186/s13062-021-00316-4 PMC8670252

[39] Angermayr SA, et al. Exploring metabolic engineering design principles for the photosynthetic production of lactic acid by *Synechocystis* sp. PCC6803. Biotechnol Biofuels. 2014;7:99. 24991233 10.1186/1754-6834-7-99 PMC4078008

[40] Kanno M, Atsumi S. Engineering an obligate photoautotrophic cyanobacterium to utilize glycerol for growth and chemical production. ACS Synth Biol. 2017;6:69–75. 27643408 10.1021/acssynbio.6b00239

[41] Angermayr SA, Hellingwerf KJ. On the use of metabolic control analysis in the optimization of cyanobacterial biosolar cell factories. J Phys Chem B. 2013;117:11169–75. 23506247 10.1021/jp4013152

