# Supplementary material for: Optimising environmental factors for maximal lactate productivity in Synechocystis sp. PCC 6803 through a design of experiments approach
Source: Biotechnol Biofuels Bioprod. 2025 Nov 25;18:118. doi: 10.1186/s13068-025-02718-2 (PMC12645717; doi:10.1186/s13068-025-02718-2)
Supplement: Supplementary file 1 — Supplementary Material 1. [file 13068_2025_2718_MOESM1_ESM.docx]

# Supplementary File 1: Optimising Environmental Factors for Maximal Lactate Productivity in *Synechocystis* sp. PCC 6803 through a Design of Experiments Approach

Contents

Supplementary Figure 1 - Sealed and non-sealed flasks – page 2

Supplementary Figure 2 – Optimal combinations of NaHCO_3_ and HEPES – page 3

Supplementary Figure 3 - Carbon Limitation in closed flask system – page 4

Supplementary Figure 4 – Comparison of growth in sealed and open flasks – page 5

Supplementary Figure 5 – Bespoke light incubator used for DOE experiment – page 6

Supplementary Figure 6 – Effect of light intensity of growth in closed flasks – page 7

Supplementary Figure 7 – Small-scale DOE-like design for the optimisation of L-lactate – page 8

Supplementary Figure 8 – Summary and accuracy of PLSR models – page 9

Supplementary Figure 9 – Modelling of input factor variables for optimisation of output factors – page 10

Supplementary Figure 10 - Interaction profiles of all two-factor interactions for biomass accumulation in the initial screening experiment – page 11

Supplementary Figure 11 - Interaction profiles of all two-factor interactions for L-lactate titre in the initial screening experiment – page 12

Supplementary Figure 12 – Interaction profiles of all two-factor interactions for L-lactate productivity in the initial screening experiment – page 13

Supplementary Figure 13 – Significant factors for biomass accumulation, L-lactate titre and productivity – page 14

Supplementary Figure 14 – Summary and accuracy of PLSR models – page 15

Supplementary Figure 15 –Significant factors for biomass accumulation, L-lactate titre and productivity – page 16

Supplementary Figure 16 – Interaction profiles of all two-factor interactions for L-lactate productivity in the second screening experiment – page 17

Supplementary Figure 17 – Determination of bounds for glycerol in DOE – page 18

Supplementary Figure 18 – Growth and productivity in various conditions – page 19

Supplementary Figure 19 – Detection and quantification of glycerol uptake – page 20

Supplementary Figure 20 - Calibration curve for measured DCW and OD at various wavelengths – page 21

Supplementary Figure 21 – Details of 2 L photobioreactors and 5 L flask scale-up – pages 21- 22

Supplementary Figure 22 – Map of plasmid SAA023 – page 23

Supplementary Figure 23 - Confirmation of full ldh*_Ll_* integration into the genome – page 23

Supplementary Table 1 – Large full-factorial experiment screening factors that affect L-lactate synthesis and growth – pages 24-25

Supplementary Table 2 – Performance metrics of the large full-factorial experiment – pages 25-26

Supplementary Table 3 – Custom small full-factorial experiment for the optimisation of L-lactate synthesis – pages 26-27

Supplementary Table 4 - Performance metrics of the small full-factorial experiment – page 28-29

Supplementary Table 5 – Validation of DOE optimised conditions – page 29

Supplementary Table 6 - 2 L photobioreactors and 5 L flask scale-up data – pages 29-32


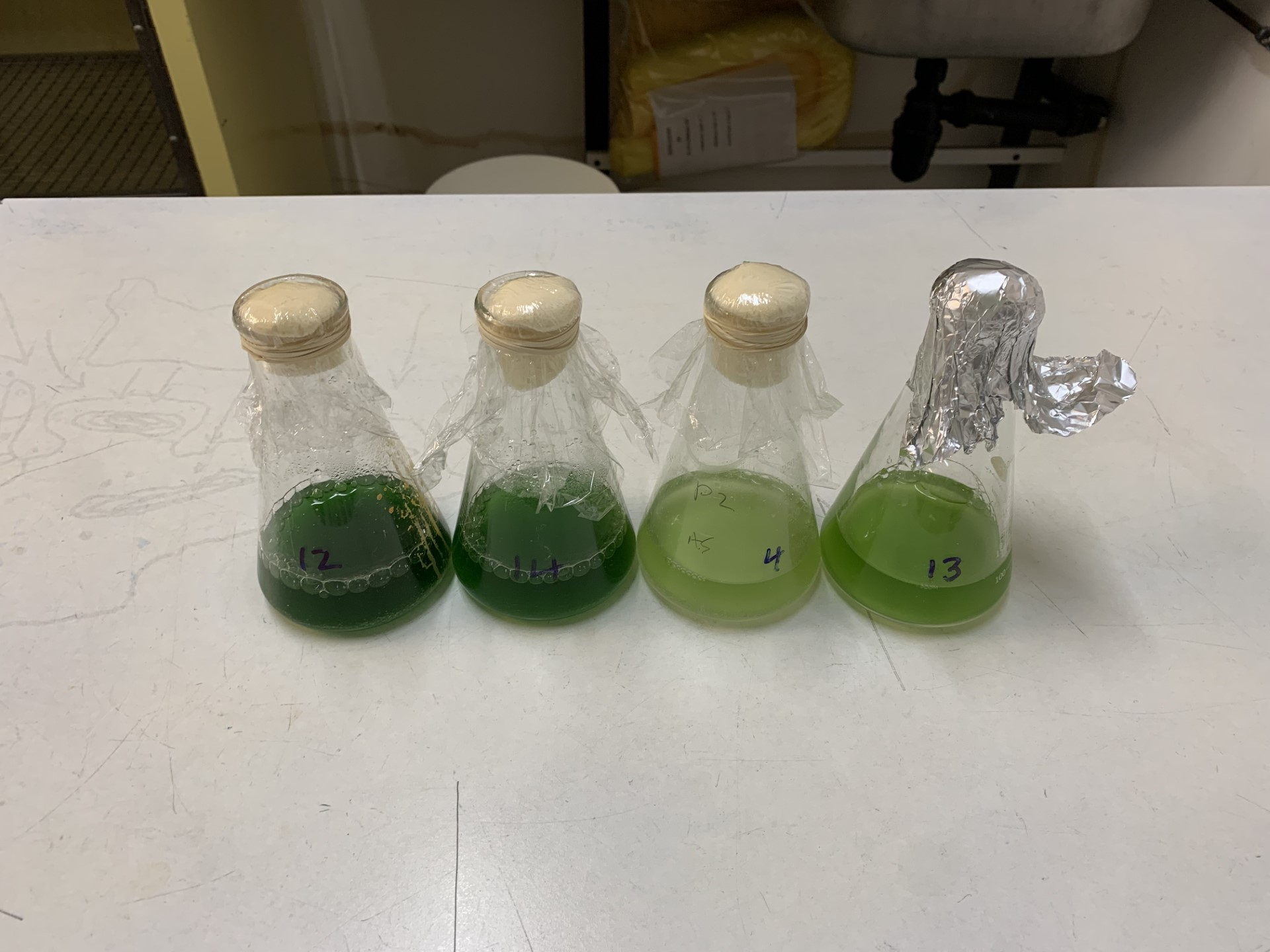


Supplementary Figure 1 – Sealed and non-sealed flasks. Left to right: Sealed flasks with 25, 12.5, 0.188 (standard BG11) mM NaHCO_3_, non-sealed flask with 0.188 (standard BG11) mM NaHCO_3_. Flasks were covered twice in Sarogold cling-film and tightened with 2 elastic bands.


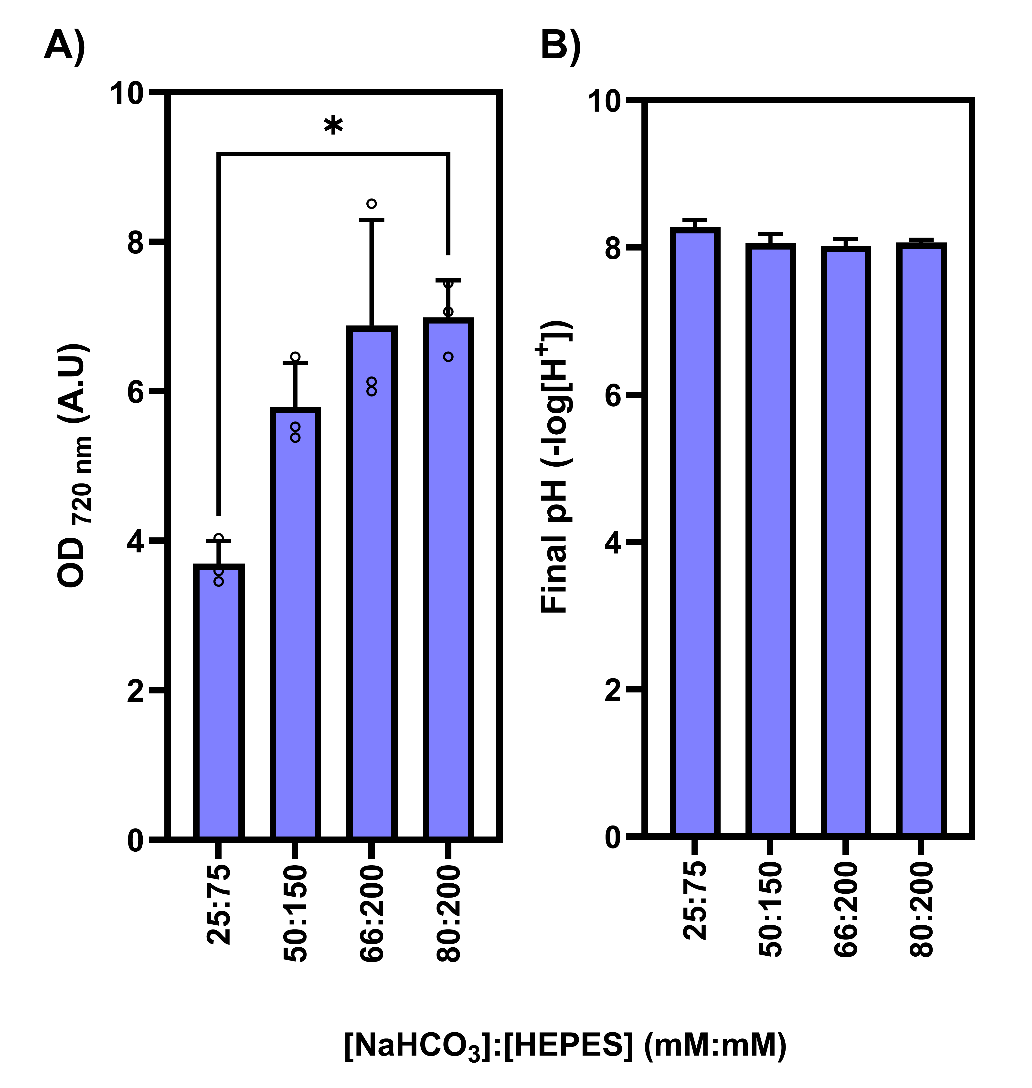


Supplementary Figure 2 – Optimal combinations of NaHCO_3_ and HEPES. Cultures were grown in BG11 media in closed flasks at 40 µmol (photons) m^-2^ s^-1^, 34 ° C, 120 rpm, from a starting OD _720 nm_ of 0.1 for 7 days. A) Culture growth as measured OD _720 nm_ as day 7. B) Culture pH measured on day 7. To prepare BG11 media, the HEPES buffer was pH corrected with 10 M KOH to pH 7.2 to start the experiment.


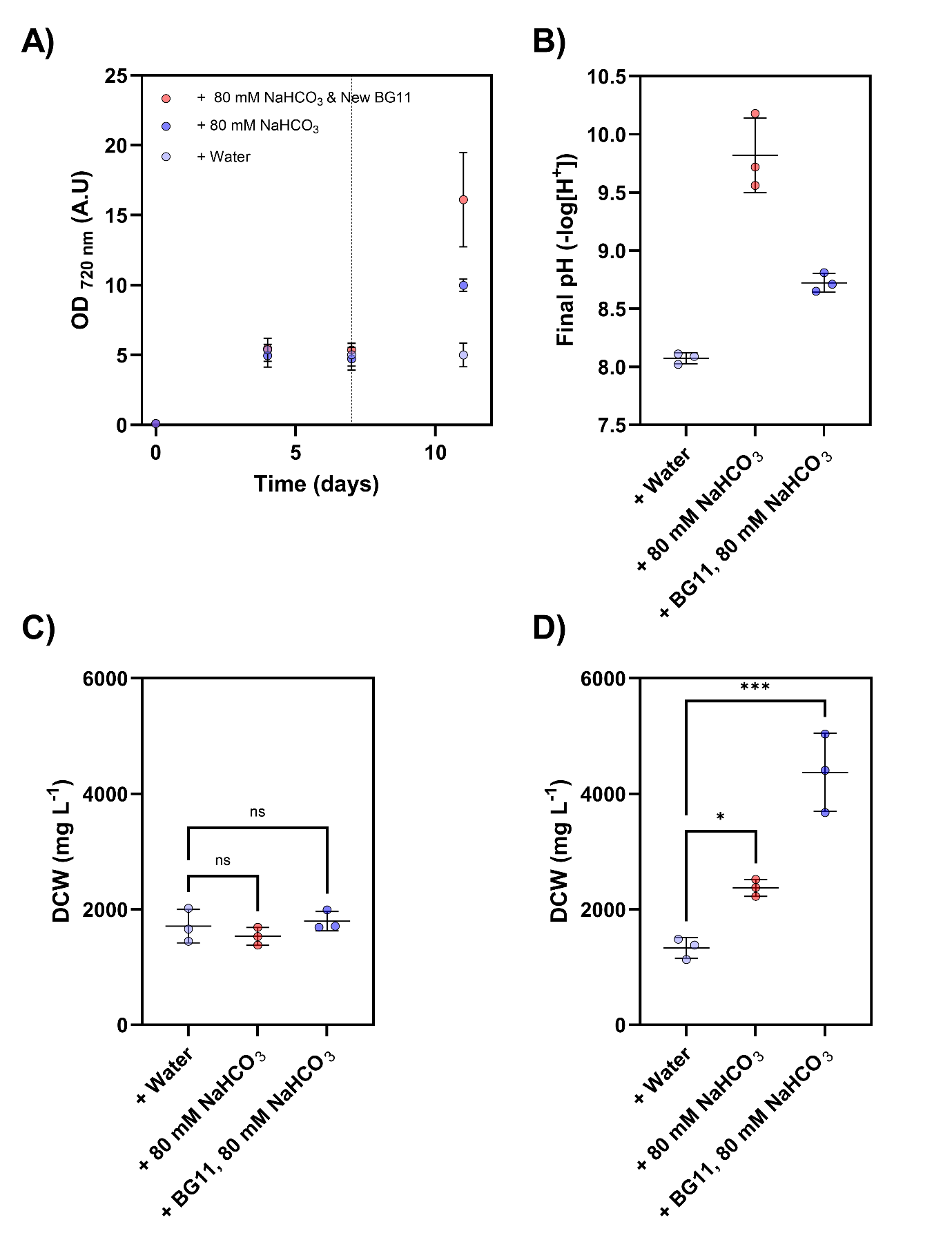


Supplementary Figure 3 - Carbon Limitation in closed flask system. Cultures were grown in BG11 media (with 80 mM NaHCO_3_ and 200 mM HEPES at pH 7.2) in closed flasks at 40 µmol (photons) m^-2^ s^-1^, 34 ° C, 120 rpm, from a starting OD _720 nm_ of 0.1 for 7 days. At day 7 the addition of either 80 mM NaHCO_3_, an equal volume of water (negative control, no more carbon but same dilution), or having the media replaced with fresh BG11 and 80 mM NaHCO_3_, was performed before 5 more days of incubation. A) Culture growth quantified by OD _720 nm_. B) Culture pH at day 12. BG11 with 200 mM HEPES was insufficient to control the media pH with a cumulative addition of 160 mM NaHCO_3_ addition. Biomass accumulation by DCW was recorded at C) day 7 (pre-additions), and D) day 12 (post-additions). One-way ANOVA test performed; ns = no significant difference, * = p < 0.05, *** = p < 0.0005. N = 3.


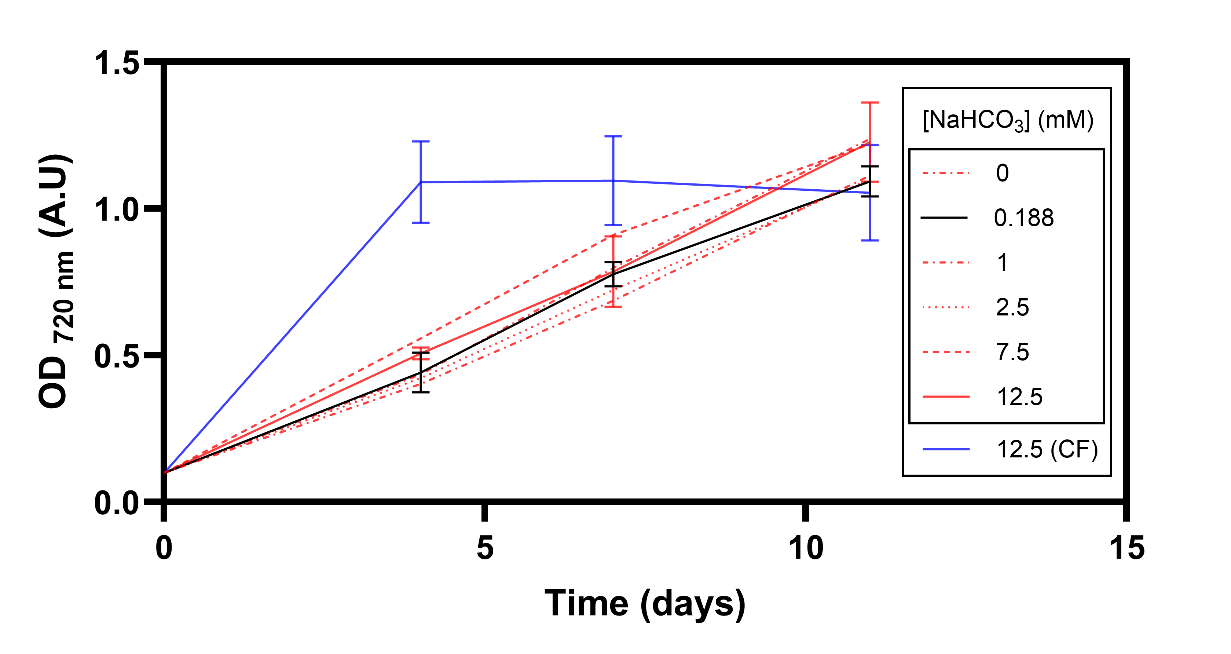


Supplementary Figure 4 – Comparison of growth in sealed and open flasks. Cling film (CF) at 12.5 mM NaHCO_3_ increased growth. N = 3 for 0, 12.5, and 12.5 mM (CF). N = 1 for 0.188 (standard BG11 recipe), 1, 2.5, and 7.5 mM. Cultures grown in BG11 media in closed flasks at 40 µmol (photons) m^-2^ s^-1^, 34 ° C, 120 rpm, from a starting OD _720 nm_ of 0.1 for 11 days.


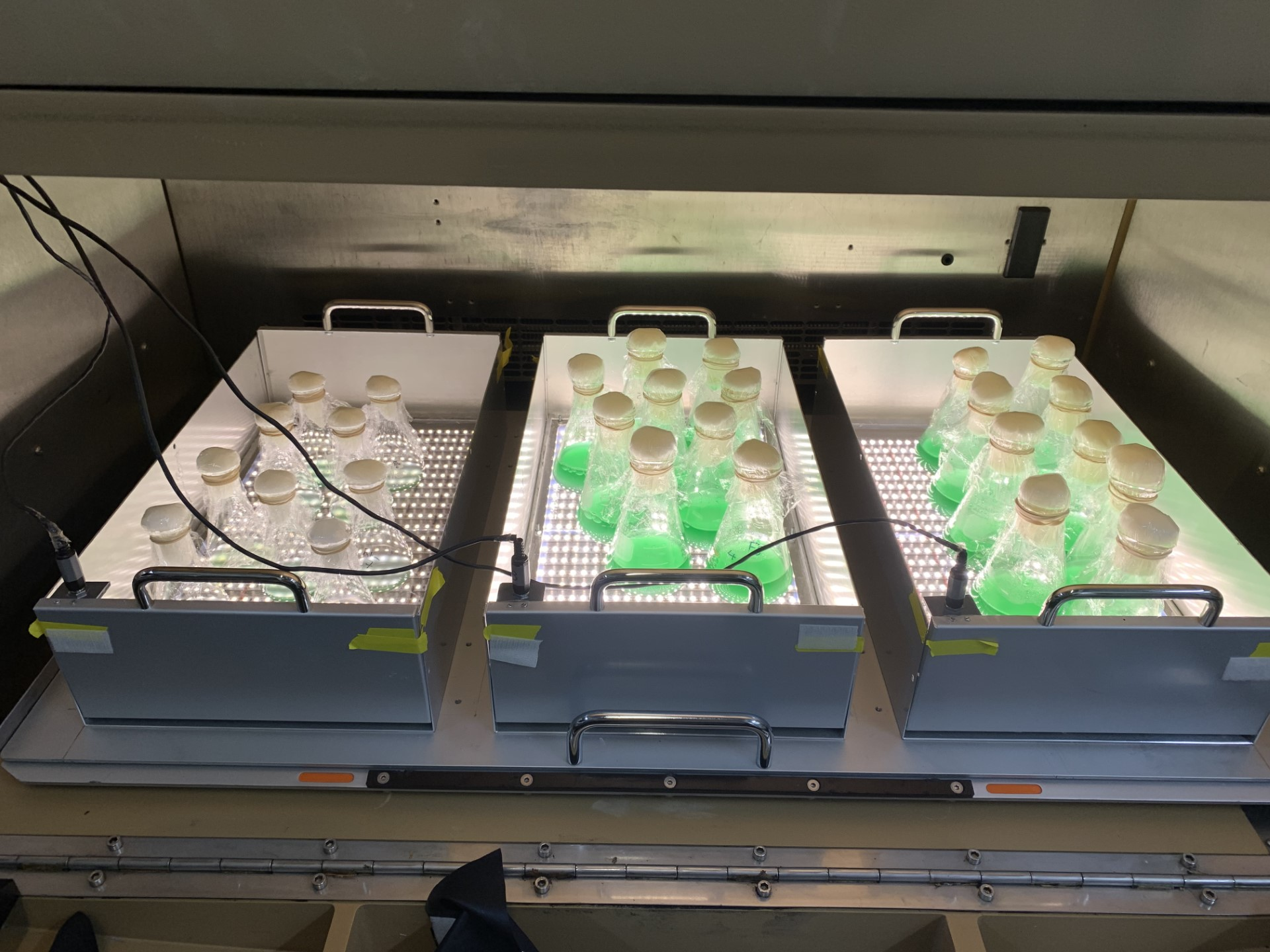


Supplementary Figure 5 – Bespoke light incubator used for DOE experiments. Lids used to cover each section and prevent light cross-over from one section to another have been removed so that cultures were visible the purpose of this photograph. With this bespoke setup we determined that different light intensities did not have a statistically significant impact on the final biomass yields of closed flasks with 80 mM HCO_3_ and 200 mM HEPES.


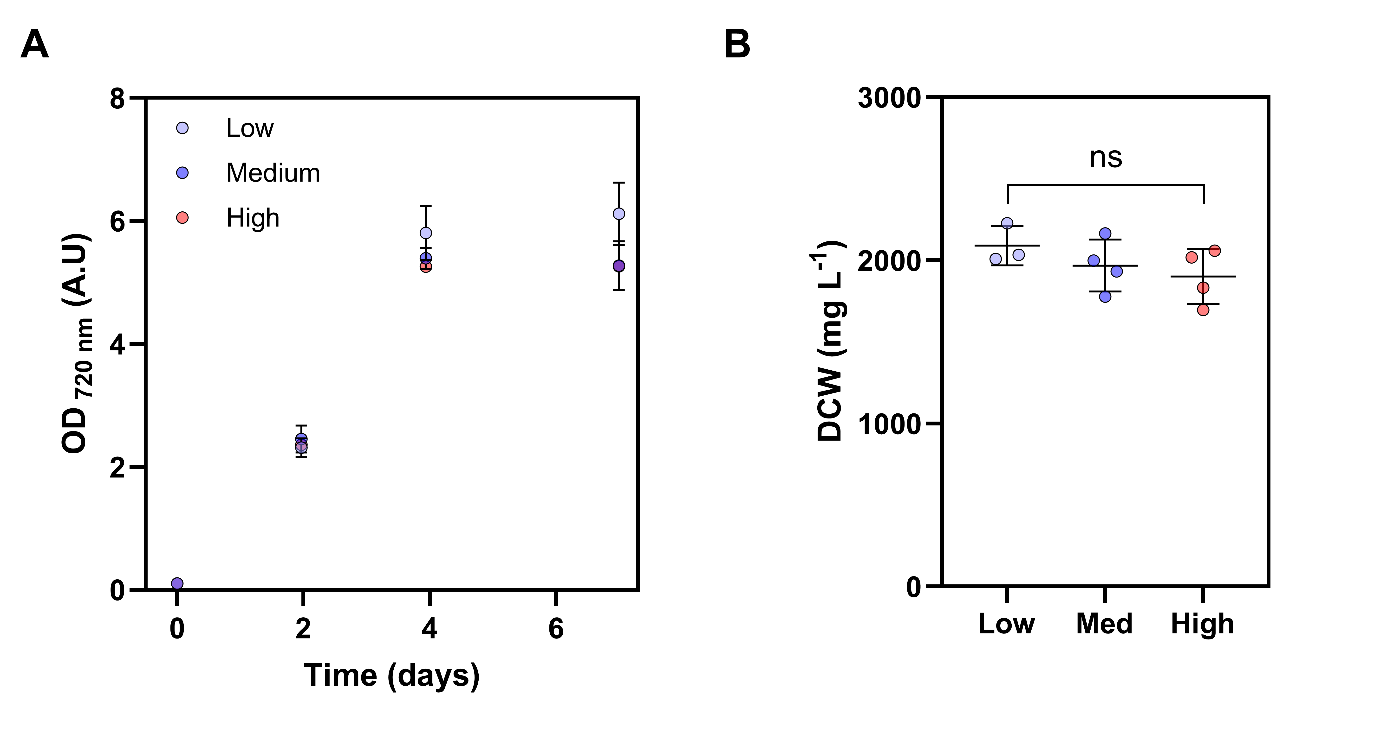


Supplementary Figure 6 – Effect of light intensity of growth in closed flasks. A) Tracking growth by increase in culture optical density. B) Biomass weight as DCW at day 7. Cultures grown in BG11 media in a closed flask at 40, 100, 200 µmol (photons) m^-2^ s^-1^, 34 ° C, 120 rpm, from a starting OD _720 nm_ of 0.1 for 4 days. N = 4.


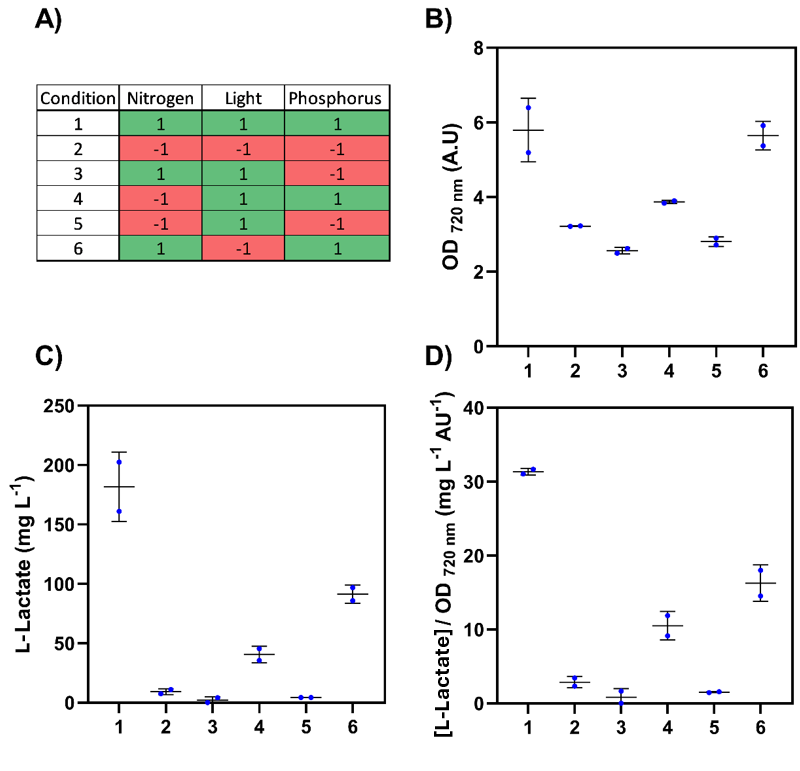


Supplementary Figure 7 – Small-scale DOE-like design for the optimisation of L-lactate. A) Table describing the media conditions for each flask. Nitrogen (NaNO_3_): 1, -1 = 17.6, 0.88 mM. Light: 1, -1 = 300, 100 µmol (photons) m^-2^ s^-1^. Phosphorus (K_2_HPO_4_): 1, -1 = 23, 0 µM. B) Culture growth as measured by optical density, C) cumulative L-lactate titre as measured by lactate assay of culture media, and D) Culture productivity as L-lactate titre normalised by culture density at day 6 of the production phase. N = 2.


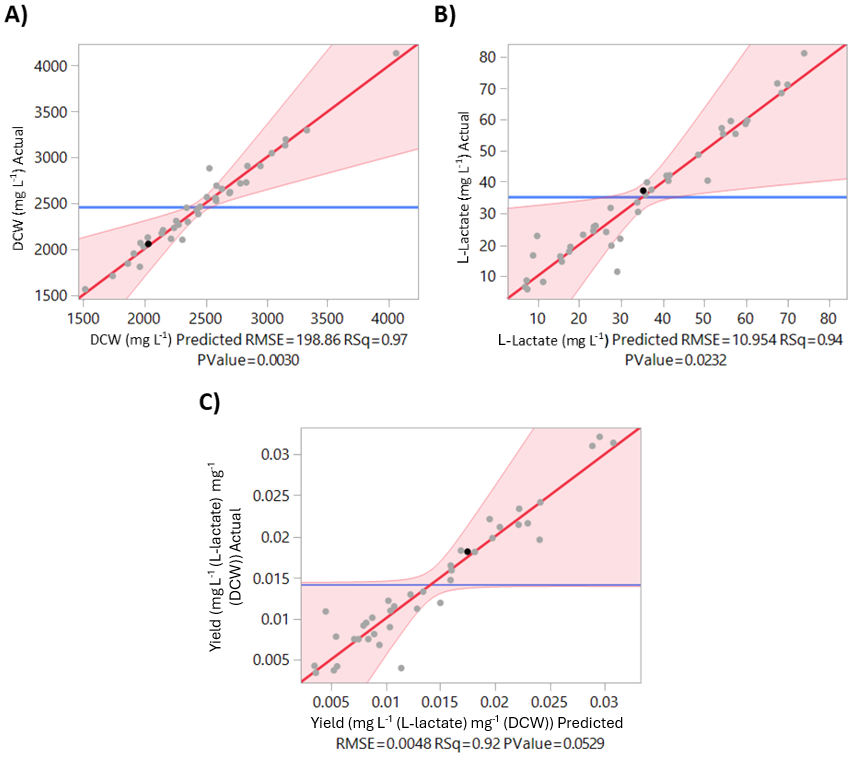


Supplementary Figure 8 – Summary and accuracy of PLSR models. Multiple PSLR models were made for A) biomass accumulation, B) Lactate titre, and C) L-lactate productivity. Input and output data from Supplementary Table 1 and Supplementary Table 2 were used to build models. Red line indicates y = x, red shaded area indicates 95 % confidence intervals. Root mean squared error (RMSE), R^2^ (RSq), and p-value (PValue) of the models are shown under the x-axis. Prediction profiler plots built in JMP 16.


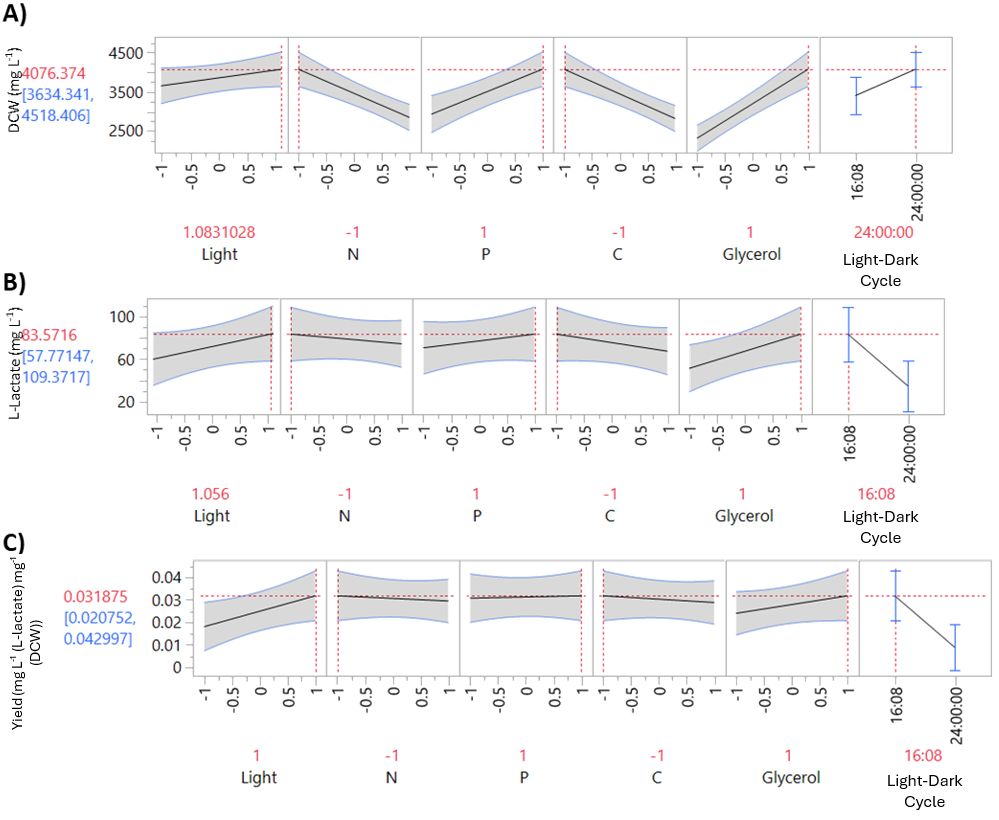


Supplementary Figure 9 – Modelling of input factor variables for optimisation of output factors. Models include A) biomass accumulation, B) Lactate titre, and C) L-lactate productivity. Red crosses in graphs indicate the factor levels predicted to optimise the specified output variables. Y axis data: numbers in red are the output variables predicted when optimised conditions are used (red crosses). Darker shaded areas indicate 95 % confidence intervals confidence intervals of line fitting. Abbreviations: N = nitrogen, P = phosphorus, C = bicarbonate.


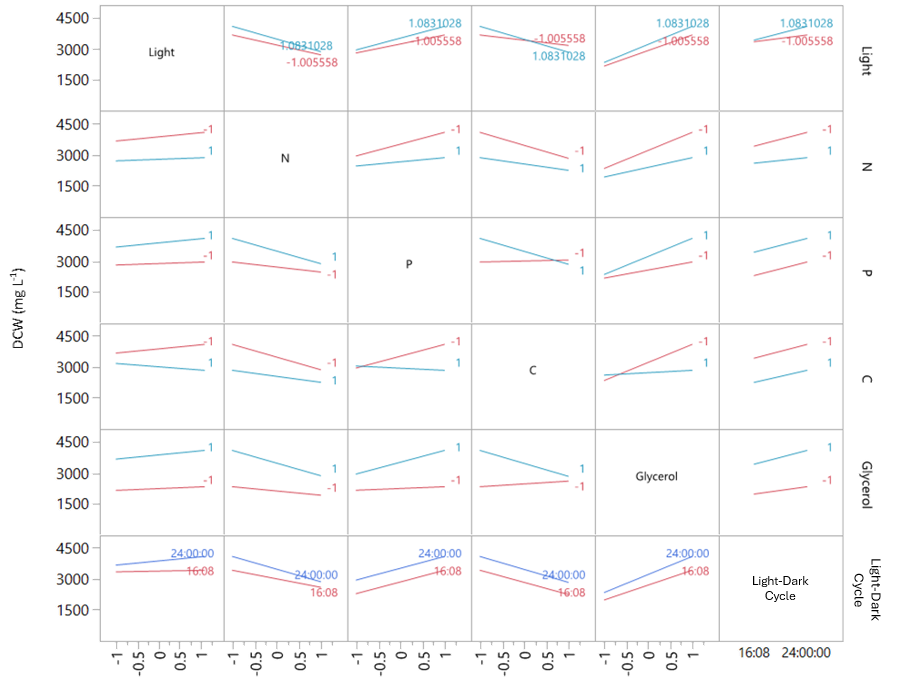


Supplementary Figure 10 – Interaction profiles of all two-factor interactions for biomass accumulation in the initial screening experiment. Nitrogen (N, NaNO_3_): 1, 0, -1 = 17.6 mM, 8.8 mM, 0.88 mM. Light: 1, -1 = 300 µmol (photons) m^-2^ s^-1^, 100 µmol (photons) m^-2^ s^-1^. Phosphorus (K_2_HPO_4_): 1, 0, -1 = 23 µM, 11.5 µM, 1.15 µM. Bicarbonate (NaHCO_3_): 1, 0, -1 = 80 mM, 60 mM, 40 mM. Glycerol: 1, 0, -1 = 50 mM, 25 mM, 0 mM. Light-Dark cycle (light:dark): 1, -1 = 24:0, 16:8. The interaction profiler plot was made in JMP 16.


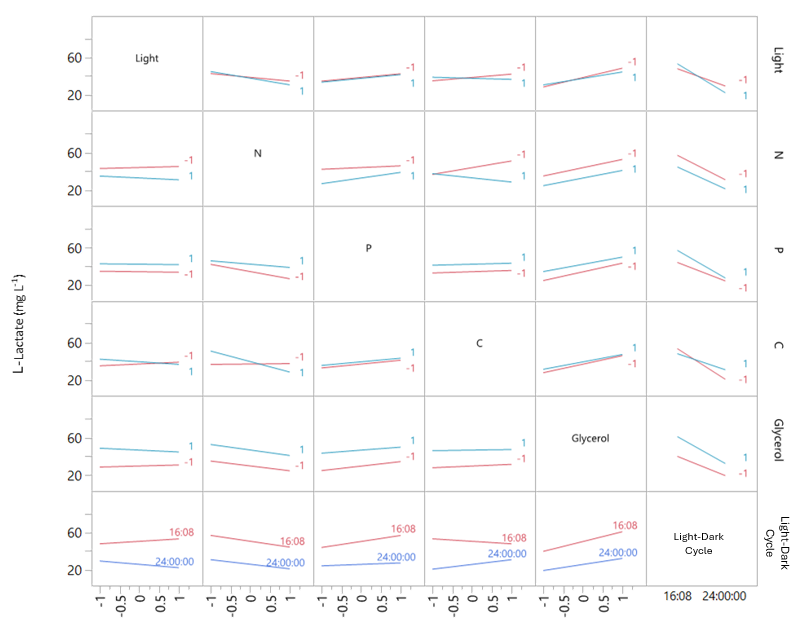


Supplementary Figure 11 - Interaction profiles of all two-factor interactions for L-lactate titre in the initial screening experiment. Nitrogen (N, NaNO_3_): 1, 0, -1 = 17.6 mM, 8.8 mM, 0.88 mM. Light: 1, -1 = 300 µmol (photons) m^-2^ s^-1^, 100 µmol (photons) m^-2^ s^-1^. Phosphorus (K_2_HPO_4_): 1, 0, -1 = 23 µM, 11.5 µM, 1.15 µM. Bicarbonate (NaHCO_3_): 1, 0, -1 = 80 mM, 60 mM, 40 mM. Glycerol: 1, 0, -1 = 50 mM, 25 mM, 0 mM. Light-Dark cycle (light:dark): 1, -1 = 24:0, 16:8. The interaction profiler plot was made in JMP 16.

**
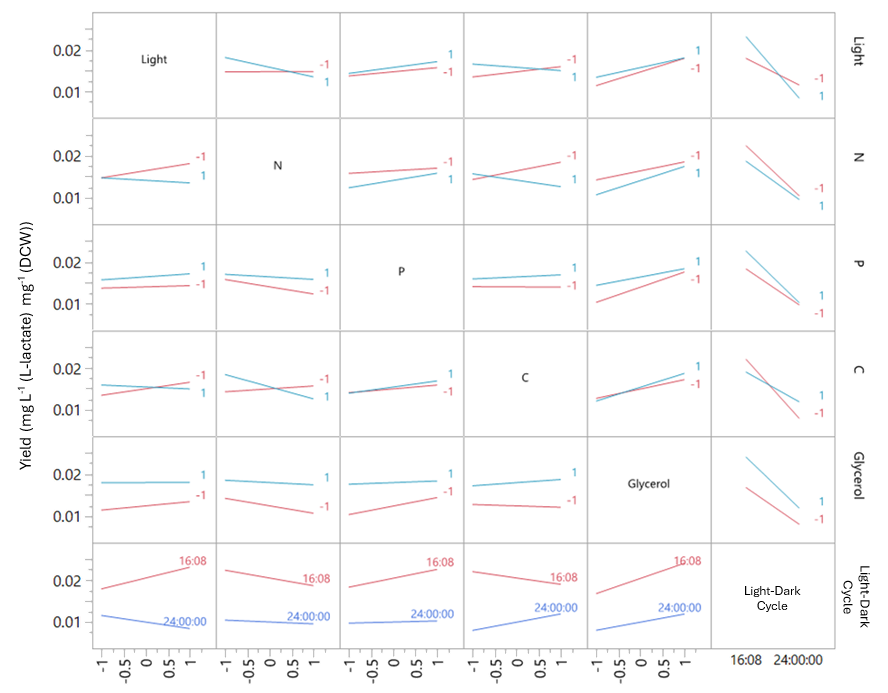
**

**Supplementary Figure 12 - Interaction profiles of all two-factor interactions for L-lactate yield in the initial screening experiment.** Nitrogen (N, NaNO_3_): 1, 0, -1 = 17.6 mM, 8.8 mM, 0.88 mM. Light: 1, -1 = 300 µmol (photons) m^-2^ s^-1^, 100 µmol (photons) m^-2^ s^-1^. Phosphorus (K_2_HPO_4_): 1, 0, -1 = 23 µM, 11.5 µM, 1.15 µM. Bicarbonate (NaHCO_3_): 1, 0, -1 = 80 mM, 60 mM, 40 mM. Glycerol: 1, 0, -1 = 50 mM, 25 mM, 0 mM. Light-Dark cycle (light:dark): 1, -1 = 24:0, 16:8. The interaction profiler plot was made in JMP 16.


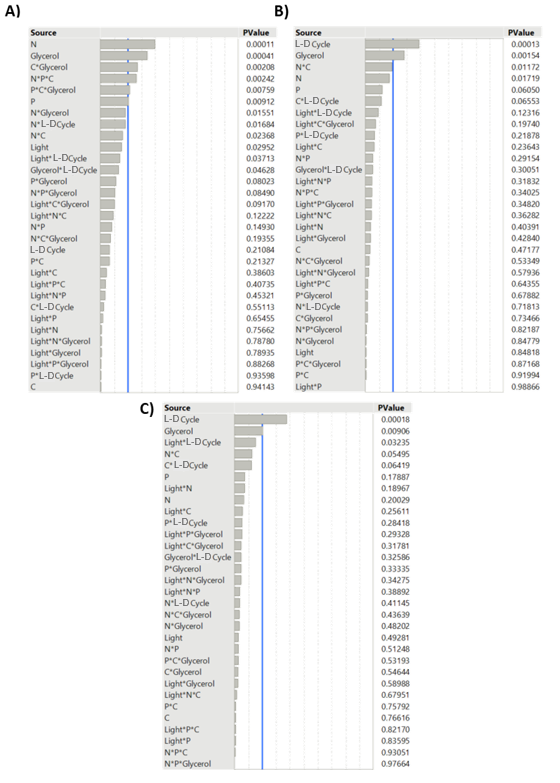


Supplementary Figure 13 – Significant factors for biomass accumulation, L-lactate titre and productivity. The blue line indicates a significant threshold at p = 0.01. Tables include PSLR models for A) biomass accumulation, B) Lactate titre, and C) L-lactate productivity. Abbreviations: N = nitrogen, P = phosphorus, C = bicarbonate.


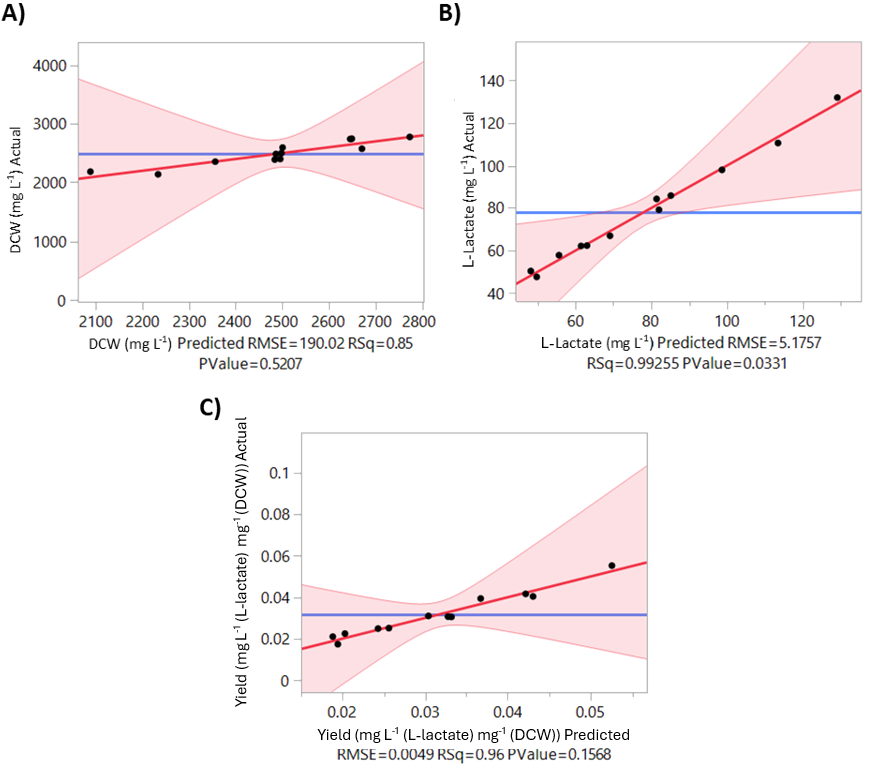


Supplementary Figure 14 - Summary and accuracy of PLSR models. Multiple PSLR models were made for A) biomass accumulation, B) Lactate titre, and C) L-lactate productivity. Input and output data from Supplementary Table 3 and Supplementary Table 4 were used to build models. Red line indicates y = x, red shaded area indicates 95 % confidence intervals. Root mean squared error (RMSE), R^2^ (RSq), and p-value (PValue) of the models are shown under the x-axis. Prediction profiler plots built in JMP 16.


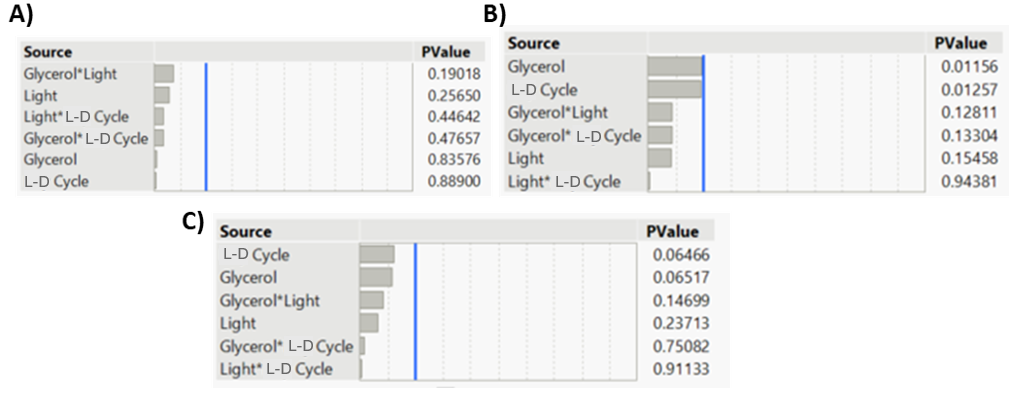


Supplementary Figure 15 – Significant factors for biomass accumulation, L-lactate titre and productivity. The blue line indicates a significant threshold at p = 0.01. Tables include PSLR models for A) biomass accumulation, B) Lactate titre, and C) L-lactate productivity.


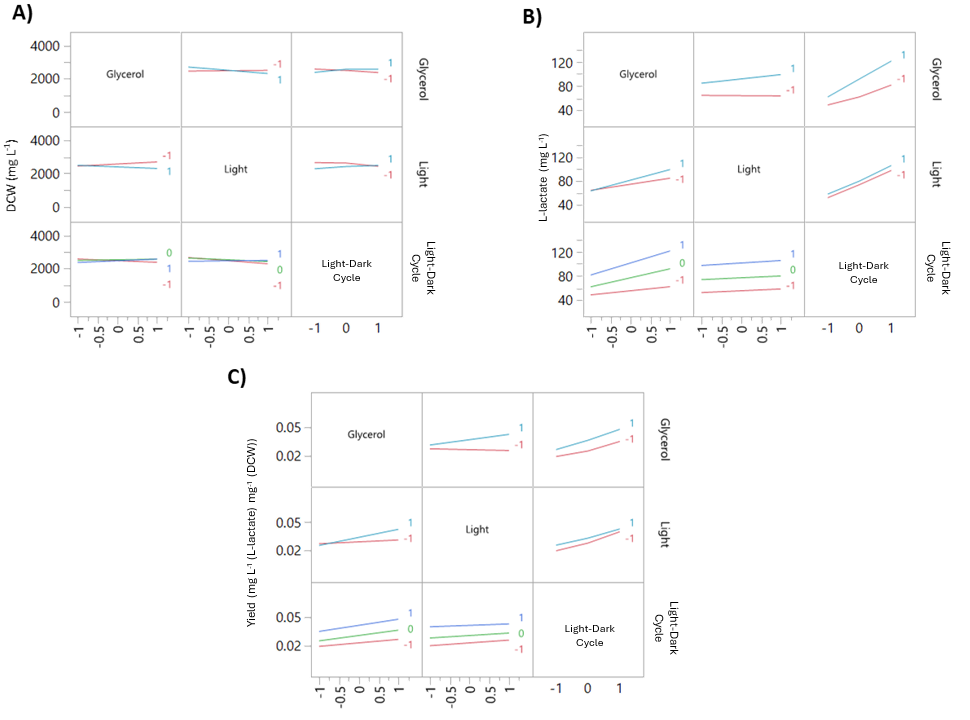


Supplementary Figure 16 - Interaction profiles of all two-factor interactions for L-lactate productivity in the second screening experiment. Light: 1, -1 = 300 µmol (photons) m^-2^ s^-1^,

100 µmol (photons) m^-2^ s^-1^. Glycerol: 1, 0, -1 = 50 mM, 25 mM, 0 mM. Day cycle (light:dark): 1, 0, -1 = 20:4, 16:8, 12:12. The interaction profiler plot was made in JMP 16.


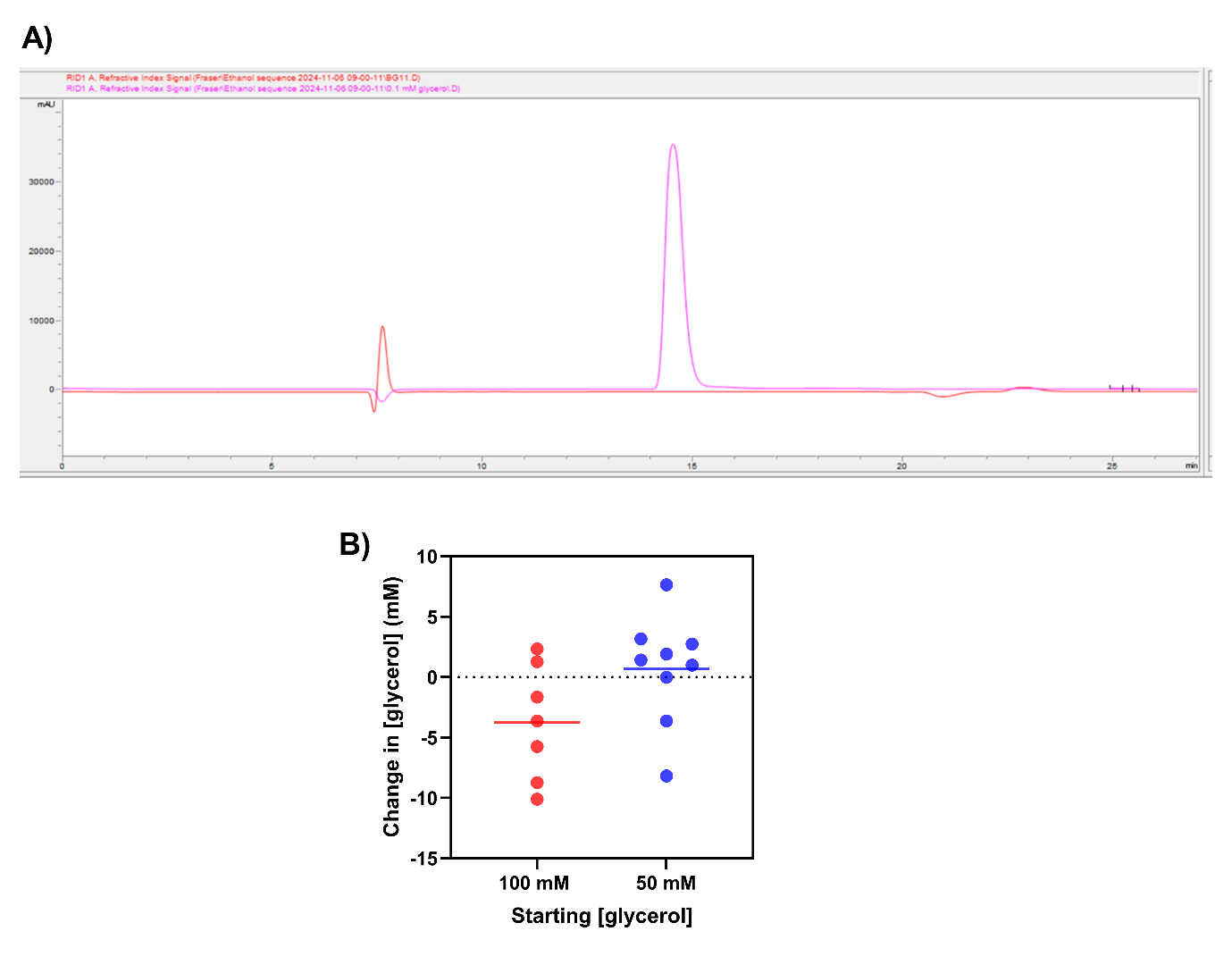


Supplementary Figure 17 – Detection and quantification of glycerol uptake. A) Exemplar HPLC trace of BG11 (red) and glycerol (pink). B) Measured glycerol consumption rates from the beginning to the end of the production phase of the optimisation experiment. Each dot represents a repeat. The lines represent the mean change in glycerol.


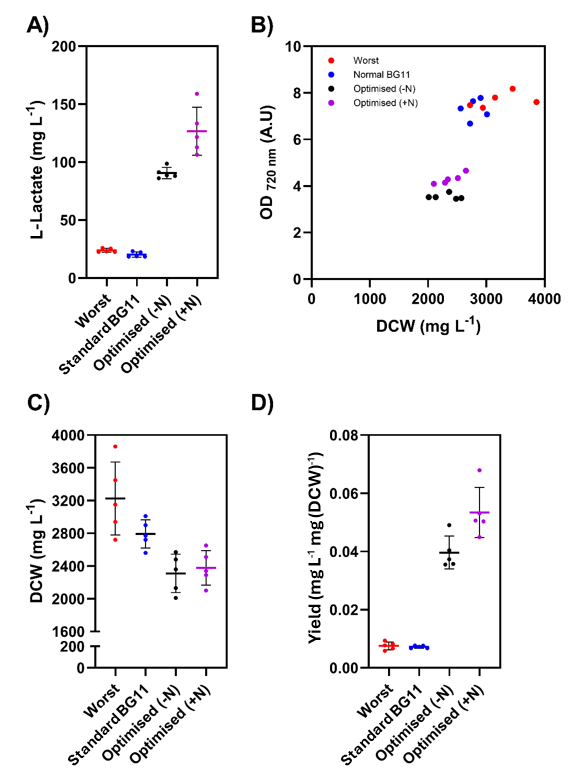


Supplementary Figure 18 – Growth and productivity in various conditions. Environmental conditions are defined in (Supplementary Table 5). Values of output variables A) L-lactate titre, B) biomass accumulation by OD _720 nm­_ and DCW, C) DCW, and D) L-lactate productivity are shown. N = 5.

Supplementary Figure 19 – Determination of bounds for glycerol in DOE. BG11 supplemented with various concentrations of glycerol grown in closed flasks at 40 µmol (photons) m^-2^ s^-1^, 34 ° C, 120 rpm, from a starting OD _720 nm_ of 0.1 for 4 days. N = 1.


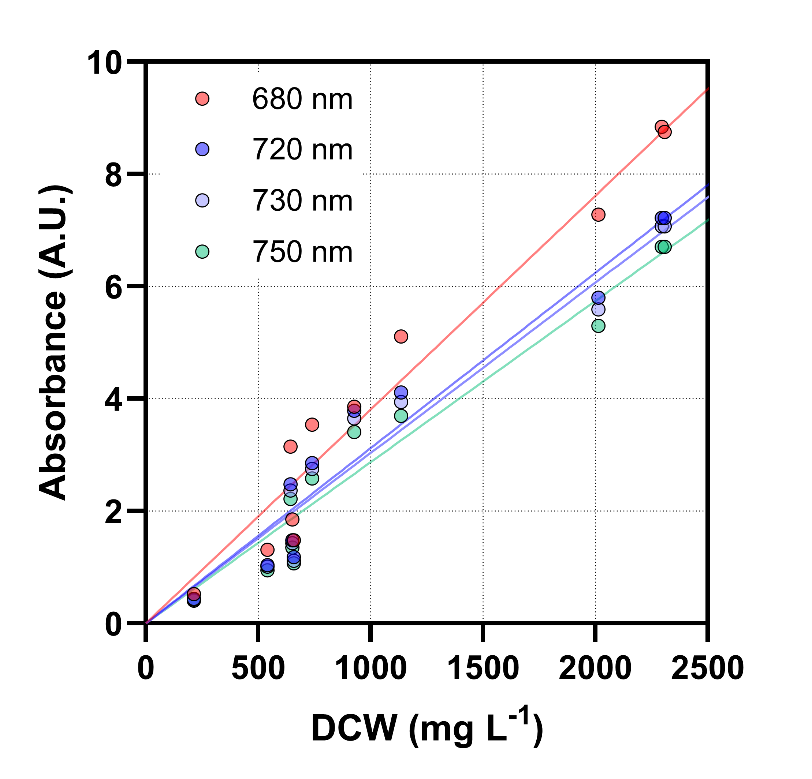


Supplementary Figure 20 – Calibration curve for measured DCW and OD at various wavelengths. Sampling was performed on cultures in a closed flask set-up.


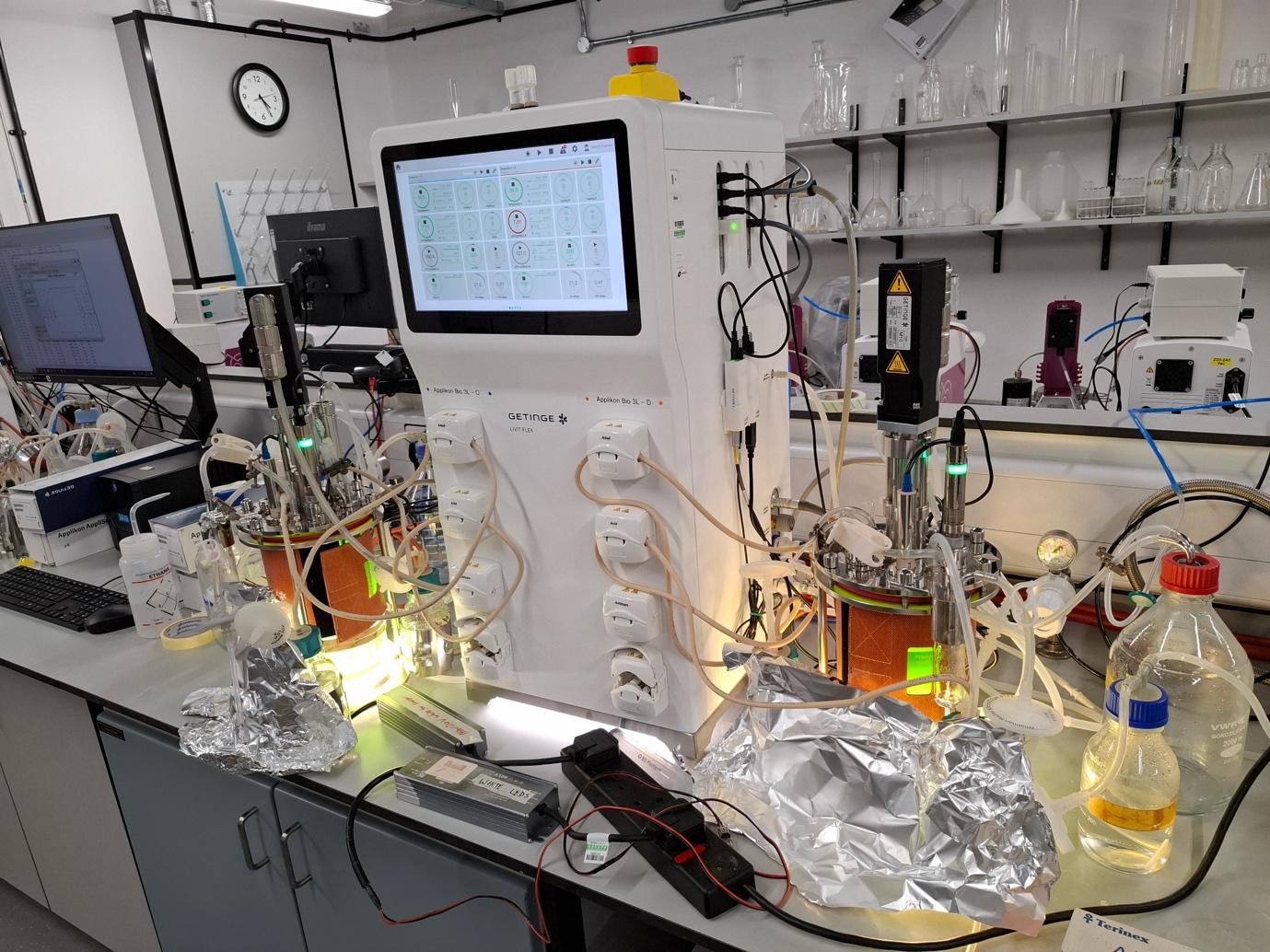


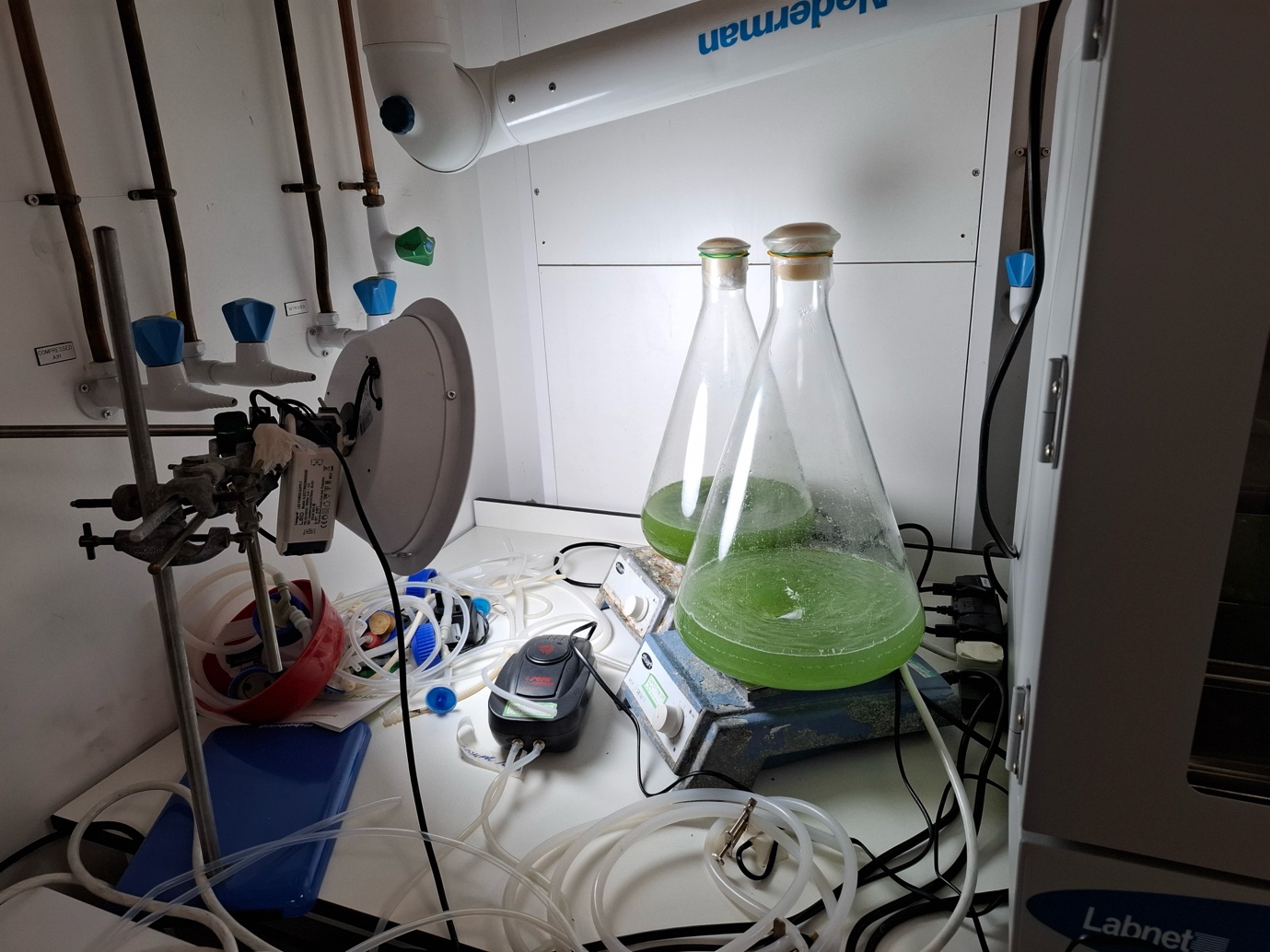


Supplementary Figure 21 – Details of 2 L photobioreactors and 5 L flask scale-up. Photographs showing the respective setups for the 2 L photobioreactor and 2 L culture in 5 L closed flasks used in this study.


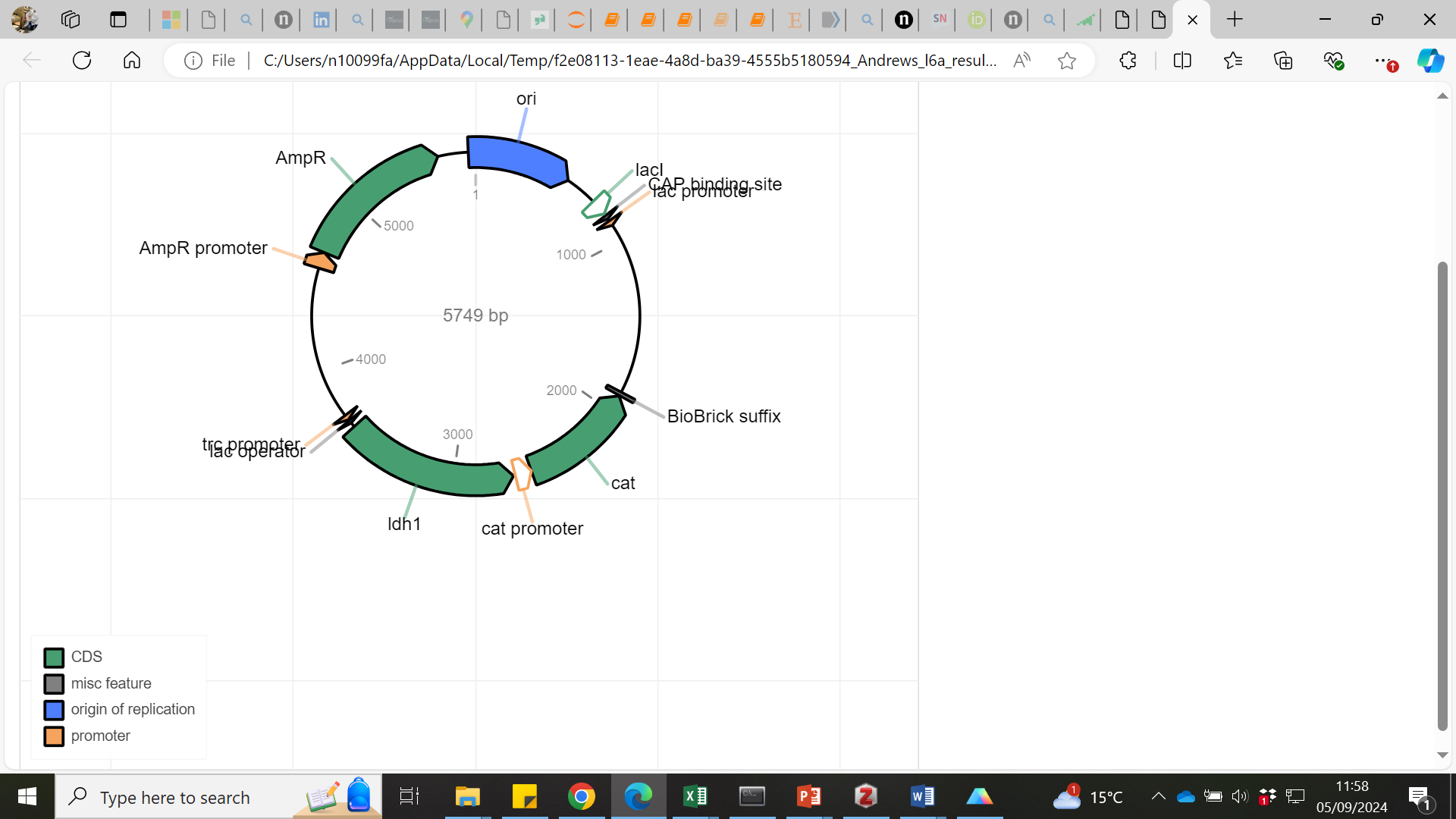


Supplementary Figure 22 - Map of plasmid SAA023. This plasmid contains the *ldh1* – L-lactate dehydrogenase with L39R substitution from *Lactococcus lactis*, trc – Ptrc promoter (hybrid between the trc and lac UV5 promoters), ori – origin of replication in E. coli, cat – Chloramphenicol resistance, lacI – Lac repressor (8 % similarity), AmpR – Ampicillin resistance. This plasmid map was produced by whole plasmid sequencing.


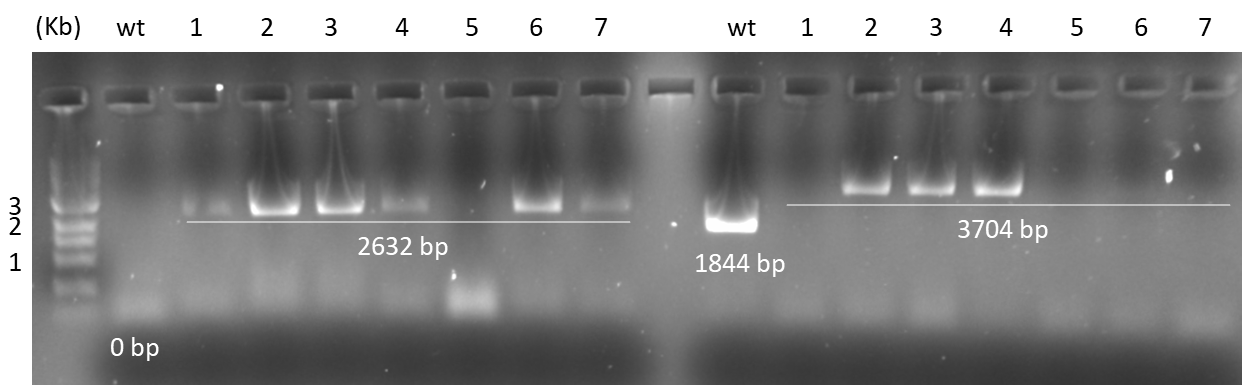


Supplementary Figure 23 - Confirmation of full ldh*_Ll_* integration into the genome. Colony PCR performed on cultures with primers that amplified between left: outside of genome integration to inside the genome integration cassette and right: across the whole genome integration site. Expected band sizes in white. No double bands on the right suggests that full genome integration has occurred.

| **Condition** | **Light** | **Nitrogen** | **Phosphorus** | **Bicarbonate** | **Glycerol** | **Light-Dark Cycle** |
| --- | --- | --- | --- | --- | --- | --- |
| 1 | 1 | 1 | -1 | -1 | 1 | 1 |
| 2 | 1 | 1 | -1 | -1 | -1 | -1 |
| 3 | -1 | 1 | -1 | -1 | 1 | 1 |
| 4 | -1 | -1 | 1 | 1 | -1 | 1 |
| 5 | 1 | -1 | -1 | -1 | 1 | -1 |
| 6 | -1 | 1 | -1 | 1 | 1 | 1 |
| 7 | -1 | 1 | -1 | -1 | 1 | -1 |
| 8 | 1 | -1 | 1 | -1 | 1 | 1 |
| 9 | -1 | 1 | 1 | 1 | 1 | -1 |
| 10 | 1 | 1 | -1 | 1 | 1 | -1 |
| 11 | -1 | 1 | -1 | -1 | -1 | 1 |
| 12 | 1 | 1 | 1 | -1 | 1 | -1 |
| 13 | -1 | 1 | 1 | 1 | -1 | 1 |
| 14 | -1 | -1 | -1 | -1 | 1 | 1 |
| 15 | 1 | -1 | 1 | -1 | -1 | -1 |
| 16 | 1 | -1 | -1 | 1 | 1 | 1 |
| 17 | 1 | 1 | 1 | 1 | -1 | -1 |
| 18 | -1 | 1 | 1 | -1 | -1 | -1 |
| 19 | 1 | 1 | 1 | -1 | 1 | 1 |
| 20 | 1 | 1 | -1 | 1 | 1 | 1 |
| 21 | 1 | 1 | 1 | 1 | 1 | 1 |
| 22 | 1 | -1 | 1 | -1 | -1 | 1 |
| 23 | -1 | -1 | 1 | 1 | 1 | 1 |
| 24 | -1 | -1 | -1 | 1 | -1 | 1 |
| 25 | -1 | 0 | 0 | 0 | 0 | 1 |
| 26 | 1 | -1 | 1 | 1 | 1 | -1 |
| 27 | 1 | 1 | 1 | -1 | -1 | 1 |
| 28 | 1 | -1 | 1 | 1 | -1 | 1 |
| 29 | -1 | 1 | -1 | 1 | -1 | 1 |
| 30 | -1 | 1 | -1 | 1 | -1 | -1 |
| 31 | -1 | 1 | 1 | -1 | 1 | 1 |
| 32 | -1 | -1 | 1 | -1 | -1 | 1 |
| 33 | 1 | -1 | 1 | 1 | 1 | 1 |
| 34 | -1 | -1 | -1 | 1 | 1 | -1 |
| 35 | -1 | -1 | -1 | -1 | -1 | -1 |
| 36 | -1 | -1 | 1 | 1 | -1 | -1 |
| 37 | 1 | -1 | -1 | 1 | -1 | -1 |
| 38 | -1 | -1 | 1 | -1 | 1 | -1 |
| 39 | 1 | -1 | -1 | -1 | -1 | 1 |
| 40 | 1 | 1 | -1 | 1 | -1 | 1 |

Supplementary Table 1 – Large full-factorial experiment screening factors that affect L-lactate synthesis and growth. Each row describes the media conditions of a flask. Nitrogen (NaNO_3_): 1, 0, -1 = 17.6, 8.8, 0.88 mM. Light: 1, -1 = 300, 100 µmol (photons) m^-2^ s^-1^. Phosphorus (K_2_HPO_4_): 1, 0, -1 = 23, 11.5, 1.15 µM. Bicarbonate (NaHCO_3_): 1, 0, -1 = 160, 120, 80 mM. Glycerol: 1, 0, -1 = 50, 25, 0 mM. Day cycle (light:dark): 1, -1 = 24:0, 16:8 hours. All conditions in single repeat.

| **Condition** | **DCW (mg L^-1^)** | **[L-lactate] (mg L^-1^)** | **[L-lactate] / DCW (mg L^-1^ mg (DCW)^-1^)** |
| --- | --- | --- | --- |
| 1 | 2380 | 17.82139 | 0.007488 |
| 2 | 2055 | 37.27398 | 0.018138 |
| 3 | 2450 | 35.95731 | 0.014676 |
| 4 | 2655 | 42.14435 | 0.015874 |
| 5 | 2265 | 71.10846 | 0.031394 |
| 6 | 1805 | 39.90692 | 0.022109 |
| 7 | 2425 | 58.5777 | 0.024156 |
| 8 | 4135 | 33.51173 | 0.008104 |
| 9 | 2565 | 55.3896 | 0.021594 |
| 10 | 1840 | 21.8961 | 0.0119 |
| 11 | 2110 | 19.30281 | 0.009148 |
| 12 | 2525 | 81.13503 | 0.032133 |
| 13 | 2295 | 23.17396 | 0.010098 |
| 14 | 2715 | 25.72902 | 0.009477 |
| 15 | 2065 | 40.46139 | 0.019594 |
| 16 | 3045 | 40.41129 | 0.013271 |
| 17 | 2555 | 42.03692 | 0.016453 |
| 18 | 2460 | 48.69675 | 0.019795 |
| 19 | 2905 | 19.69151 | 0.006778 |
| 20 | 2125 | 16.56575 | 0.007796 |
| 21 | 2230 | 24.40854 | 0.010946 |
| 22 | 2100 | 22.80667 | 0.01086 |
| 23 | 3130 | 57.20889 | 0.018278 |
| 24 | 2905 | 37.55338 | 0.012927 |
| 25 | 2880 | 11.39776 | 0.003958 |
| 26 | 2305 | 71.50597 | 0.031022 |
| 27 | 1950 | 14.6139 | 0.007494 |
| 28 | 2690 | 24.07015 | 0.008948 |
| 29 | 1560 | 6.508968 | 0.004172 |
| 30 | 2025 | 8.568948 | 0.004232 |
| 31 | 2610 | 31.74424 | 0.012163 |
| 32 | 2170 | 16.24522 | 0.007486 |
| 33 | 2725 | 30.47693 | 0.011184 |
| 34 | 3195 | 68.44054 | 0.021421 |
| 35 | 2270 | 26.05174 | 0.011477 |
| 36 | 2620 | 55.37845 | 0.021137 |
| 37 | 2545 | 59.4829 | 0.023372 |
| 38 | 3295 | 59.66589 | 0.018108 |
| 39 | 2205 | 8.12158 | 0.003683 |
| 40 | 1705 | 5.773711 | 0.003386 |

Supplementary Table 2 – Performance metrics of the large full-factorial experiment. Conditions used for L-lactate production are specified in Supplementary Table 1.

| **Condition** | **Glycerol** | **Light** | **Light-Dark Cycle** |
| --- | --- | --- | --- |
| 1 | -1 | -1 | -1 |
| 2 | 1 | -1 | -1 |
| 3 | -1 | 1 | -1 |
| 4 | 1 | 1 | -1 |
| 5 | -1 | -1 | 0 |
| 6 | 1 | -1 | 0 |
| 7 | -1 | 1 | 0 |
| 8 | 1 | 1 | 0 |
| 9 | -1 | -1 | 1 |
| 10 | 1 | -1 | 1 |
| 11 | -1 | 1 | 1 |
| 12 | 1 | 1 | 1 |

Supplementary Table 3 - Custom small full-factorial experiment for the optimisation of L-lactate synthesis. Each row describes the media conditions of a flask. Light: 1, -1 = 300, 100 µmol (photons) m^-2^ s^-1^. Glycerol: 1, -1 = 100, 50 mM. Day cycle (light:dark): 1, 0, -1 = 12:12, 16:8, 20:4 hours. All conditions in single repeat.

| **Condition** | **DCW (mg L^-1^)** | **[L-lactate] (mg L^-1^ )** | **[L-lactate] / DCW (mg L^-1^ mg (DCW)^-1^)** |
| --- | --- | --- | --- |
| 1 | 2745 | 47.58280644 | 0.017334356 |
| 2 | 2575 | 57.79538418 | 0.022444809 |
| 3 | 2400 | 50.34735774 | 0.020978066 |
| 4 | 2185 | 66.93466552 | 0.030633714 |
| 5 | 2485 | 62.31623865 | 0.025076957 |
| 6 | 2775 | 85.86371087 | 0.030941878 |
| 7 | 2500 | 62.15361798 | 0.024861447 |
| 8 | 2355 | 97.96268831 | 0.041597745 |
| 9 | 2140 | 84.27002836 | 0.039378518 |
| 10 | 2740 | 110.5820519 | 0.040358413 |
| 11 | 2595 | 79.16373949 | 0.030506258 |
| 12 | 2390 | 131.9829313 | 0.055222984 |

Supplementary Table 4 - Performance metrics of the small full-factorial experiment. Conditions used for L-lactate production are specified in Supplementary Table 3.

|  | **Light** | **Nitrogen** | **Phosphorus** | **Glycerol** | **Light-Dark Cycle** |
| --- | --- | --- | --- | --- | --- |
| Standard | -1 | 1 | 1 | -1 | -1 |
| Worst | -1 | 1 | -1 | -1 | -1 |
| Optimised (+N) | 1 | 1 | 1 | 1 | 1 |
| Optimised (-N) | 1 | -1 | 1 | 1 | 1 |

Supplementary Table 5 – Validation of DOE optimised conditions. Each row describes the media conditions of a flask. Light: 1, -1 = 300, 100 µmol (photons) m^-2^ s^-1^. Glycerol: 1, -1 = 100, 0 mM. Day cycle (light:dark): 1, -1 = 12:12, 24:0 hours. Nitrogen (NaNO_3_): 1, -1 = 17.6, 0.88 mM. Phosphorus (K_2_HPO_4_): 1, 0, -1 = 23, 11.5, 1.15 µM. N = 5.

Supplementary Table 6 - 2 L photobioreactors and 5 L flask scale-up data. Photobioreactors 1&2 and Flasks 1&2 are duplicate repeats of the DOE optimum conditions and Photobioreactors 3&4 and Flasks 3&4 are duplicate repeats of the pre-DOE initial conditions. OD720, OD680, Cell Dry Weight (CDW) and lactate titre were all measured directly, whereas yield and productivity were calculated by dividing the titre by the CDW for yield then dividing by the time elapsed for product

| **Vessel** | **hours elapsed** | **OD720**  **(A.U)** | **OD680**  **(A.U)** | **CDW**  **(mg)** | **Lactate titre (mg)** | **Productivity**  **(mg lactate / mg CDW)** | **Rate**  **(mg lactate / mg CDW / hour)** |
| --- | --- | --- | --- | --- | --- | --- | --- |
| Photobioreactor 1 | 0.00 | 0.4 | 0.5 | 228 | 0 | 0.00000 | 0.00000 |
|  | 5.50 | 1.8 | 2.0 | 492 | 0 | 0.00000 | 0.00000 |
|  | 22.55 | 5.1 | 5.6 | 1393 | 6.4 | 0.00459 | 0.00020 |
|  | 30.00 | 6.3 | 6.9 | 1720 | 11.2 | 0.00651 | 0.00022 |
|  | 46.75 | 8.6 | 9.3 | 2326 | 24.9 | 0.01071 | 0.00023 |
|  | 54.25 | 9.0 | 9.8 | 2451 | 28.1 | 0.01146 | 0.00021 |
|  | 70.00 | 10.6 | 11.5 | 2884 | 33.8 | 0.01172 | 0.00017 |
|  | 77.25 | 11.0 | 11.9 | 2979 | 40.7 | 0.01366 | 0.00018 |
|  | 95.85 | 11.5 | 12.5 | 3117 | 50.1 | 0.01607 | 0.00017 |
|  | 97.25 | 10.0 | 11.3 | 3125 | 51.6 | 0.01651 | 0.00017 |
| Photobioreactor 2 | 0.00 | 0.4 | 0.5 | 228 | 0 | 0.00000 | 0.00000 |
|  | 5.50 | 1.4 | 1.6 | 394 | 0 | 0.00000 | 0.00000 |
|  | 22.55 | 3.2 | 3.5 | 867 | 7.2 | 0.00830 | 0.00037 |
|  | 30.00 | 3.7 | 4.0 | 993 | 8.5 | 0.00856 | 0.00029 |
|  | 46.75 | 6.4 | 6.9 | 1735 | 27.4 | 0.01579 | 0.00034 |
|  | 54.25 | 6.9 | 7.5 | 1881 | 31.8 | 0.01691 | 0.00031 |
|  | 70.00 | 9.8 | 10.6 | 2659 | 41.9 | 0.01576 | 0.00023 |
|  | 77.25 | 10.4 | 11.3 | 2814 | 46.1 | 0.01638 | 0.00021 |
|  | 95.85 | 10.4 | 12.2 | 3278 | 58.4 | 0.01782 | 0.00019 |
|  | 97.25 | 11.0 | 12.5 | 3390 | 60.6 | 0.01788 | 0.00018 |
| Photobioreactor 3 | 0.00 | 0.4 | 0.5 | 241 | 0 | 0.00000 | 0.00000 |
|  | 4.50 | 1.2 | 1.3 | 332 | 0 | 0.00000 | 0.00000 |
|  | 20.50 | 2.8 | 3.0 | 749 | 2.3 | 0.00307 | 0.00015 |
|  | 27.45 | 3.0 | 3.3 | 824 | 5.2 | 0.00631 | 0.00023 |
|  | 44.75 | 7.0 | 7.6 | 1905 | 12.6 | 0.00661 | 0.00015 |
|  | 52.25 | 8.2 | 8.9 | 2217 | 15.8 | 0.00713 | 0.00014 |
|  | 69.00 | 10.1 | 11.0 | 2753 | 17.1 | 0.00621 | 0.00009 |
|  | 76.00 | 10.8 | 11.8 | 2938 | 20.5 | 0.00698 | 0.00009 |
|  | 92.50 | 12.6 | 13.7 | 3426 | 28.9 | 0.00844 | 0.00009 |
|  | 98.00 | 12.6 | 14.9 | 3552 | 30.4 | 0.00856 | 0.00009 |
| Photobioreactor 4 | 0.00 | 0.4 | 0.5 | 241 | 0 | 0.00000 | 0.00000 |
|  | 4.50 | 1.4 | 1.5 | 386 | 0 | 0.00000 | 0.00000 |
|  | 20.50 | 2.9 | 3.2 | 801 | 3.5 | 0.00437 | 0.00021 |
|  | 27.45 | 3.5 | 3.9 | 963 | 7.9 | 0.00820 | 0.00030 |
|  | 44.75 | 7.2 | 7.8 | 1952 | 10.7 | 0.00548 | 0.00012 |
|  | 52.25 | 7.6 | 8.3 | 2064 | 12.2 | 0.00591 | 0.00011 |
|  | 69.00 | 9.3 | 10.2 | 2539 | 16.3 | 0.00642 | 0.00009 |
|  | 76.00 | 10.5 | 11.4 | 2841 | 19.8 | 0.00697 | 0.00009 |
|  | 92.50 | 12.5 | 13.6 | 3395 | 24 | 0.00707 | 0.00008 |
|  | 98.00 | 11.6 | 13.9 | 3417 | 25.2 | 0.00737 | 0.00008 |
| Flask 1 | 0 | 0.4 | 0.5 | 217 | 0 | 0.00000 | 0.00000 |
|  | 5.5 | 1.1 | 1.2 | 291 | 0 | 0.00000 | 0.00000 |
|  | 20.75 | 2.5 | 2.8 | 691 | 5.3 | 0.01821 | 0.00088 |
|  | 29 | 3.5 | 3.8 | 962 | 18.5 | 0.01923 | 0.00066 |
|  | 45.25 | 5.1 | 5.6 | 1388 | 44.1 | 0.03177 | 0.00070 |
|  | 53.5 | 6.3 | 6.9 | 1715 | 56 | 0.03265 | 0.00061 |
|  | 69.5 | 9.2 | 10.0 | 2509 | 71.4 | 0.02846 | 0.00041 |
|  | 77.25 | 9.8 | 10.7 | 2673 | 80.2 | 0.03000 | 0.00039 |
|  | 92.75 | 10.5 | 11.4 | 2840 | 92.7 | 0.03264 | 0.00035 |
|  | 98 | 10.5 | 11.4 | 2854 | 95.3 | 0.03339 | 0.00034 |
| Flask 2 | 0 | 0.4 | 0.5 | 217 | 0 | 0.00000 | 0.00000 |
|  | 5.5 | 1.0 | 1.0 | 261 | 0 | 0.00000 | 0.00000 |
|  | 20.75 | 2.0 | 2.2 | 542 | 0 | 0.00000 | 0.00000 |
|  | 29 | 2.9 | 3.2 | 798 | 13.8 | 0.01729 | 0.00060 |
|  | 45.25 | 4.6 | 5.0 | 1253 | 32.9 | 0.02626 | 0.00058 |
|  | 53.5 | 5.5 | 6.0 | 1502 | 40.3 | 0.02683 | 0.00050 |
|  | 69.5 | 7.9 | 8.5 | 2137 | 57.1 | 0.02672 | 0.00038 |
|  | 77.25 | 9.0 | 9.8 | 2449 | 65.4 | 0.02670 | 0.00035 |
|  | 92.75 | 9.9 | 10.7 | 2684 | 74.6 | 0.02779 | 0.00030 |
|  | 98 | 10.0 | 10.9 | 2716 | 80.4 | 0.02960 | 0.00030 |
| Flask 3 | 0 | 0.4 | 0.5 | 240 | 0 | 0.00000 | 0.00000 |
|  | 5.5 | 0.9 | 1.0 | 251 | 0 | 0.00000 | 0.00000 |
|  | 20.75 | 1.1 | 1.2 | 309 | 3.9 | 0.01262 | 0.00061 |
|  | 29 | 1.4 | 1.6 | 389 | 7.2 | 0.01851 | 0.00064 |
|  | 45.25 | 3.5 | 3.9 | 963 | 16.4 | 0.01703 | 0.00038 |
|  | 53.5 | 4.5 | 4.9 | 1224 | 27.3 | 0.02230 | 0.00042 |
|  | 69.5 | 7.7 | 8.4 | 2090 | 32.6 | 0.01560 | 0.00022 |
|  | 77.25 | 8.6 | 9.4 | 2342 | 36.1 | 0.01541 | 0.00020 |
|  | 92.75 | 9.6 | 10.5 | 2618 | 41.8 | 0.01597 | 0.00017 |
|  | 98 | 10.4 | 11.4 | 2838 | 45 | 0.01586 | 0.00016 |
| Flask 4 | 0 | 0.4 | 0.5 | 240 | 0 | 0.00000 | 0.00000 |
|  | 5.5 | 0.9 | 1.0 | 251 | 0 | 0.00000 | 0.00000 |
|  | 20.75 | 1.2 | 1.3 | 334 | 0 | 0.00000 | 0.00000 |
|  | 29 | 1.5 | 1.6 | 402 | 4.2 | 0.01045 | 0.00036 |
|  | 45.25 | 3.0 | 3.2 | 805 | 9.8 | 0.01217 | 0.00027 |
|  | 53.5 | 3.7 | 4.1 | 1017 | 13.9 | 0.01367 | 0.00026 |
|  | 69.5 | 7.2 | 7.8 | 1956 | 16.5 | 0.00844 | 0.00012 |
|  | 77.25 | 8.4 | 9.1 | 2277 | 20.7 | 0.00909 | 0.00012 |
|  | 92.75 | 9.8 | 10.6 | 2650 | 30.1 | 0.01136 | 0.00012 |
|  | 98 | 11.0 | 12.0 | 2996 | 35.6 | 0.01188 | 0.00012 |
